# Bayesian Regression Model for a Cost-Utility and Cost-Effectiveness Analysis Comparing Punch Grafting Versus Usual Care for the Treatment of Chronic Wounds

**DOI:** 10.3390/ijerph17113823

**Published:** 2020-05-28

**Authors:** Carmen Selva-Sevilla, Elena Conde-Montero, Manuel Gerónimo-Pardo

**Affiliations:** 1 Department of Applied Economy, Facultad de Ciencias Económicas y Empresariales de Albacete, Universidad de Castilla La Mancha, Plaza de la Universidad 1, 02071 Albacete, Spain; 2 Department of Dermatology, Hospital Universitario Infanta Leonor, Avenida Gran Vía del Este 80, 28031 Madrid, Spain; elenacondemontero@gmail.com; 3 Department of Anesthesiology, Complejo Hospitalario Universitario de Albacete, Calle Hermanos Falcó 37, 02006 Albacete, Spain; sergepu@hotmail.com

**Keywords:** health economics, economic evaluation, health outcomes, health care, quality-adjusted life years, EuroQoL-5D, sensitivity analysis

## Abstract

Punch grafting is a traditional technique used to promote epithelialization of hard-to-heal wounds. The main purpose of this observational study was to conduct a cost-utility analysis (CUA) and a cost-effectiveness analysis (CEA) comparing punch grafting (*n* = 46) with usual care (*n* = 34) for the treatment of chronic wounds in an outpatient specialized wound clinic from a public healthcare system perspective (Spanish National Health system) with a three-month time horizon. CUA outcome was quality-adjusted life years (QALYs) calculated from EuroQoL-5D, whereas CEA outcome was wound-free period. One-way sensitivity analyses, extreme scenario analysis, and re-analysis by subgroups were conducted to fight against uncertainty. Bayesian regression models were built to explore whether differences between groups in costs, wound-free period, and QALYs could be explained by other variables different to treatment. As main results, punch grafting was associated with a reduction of 37% in costs compared to usual care, whereas mean incremental utility (0.02 ± 0.03 QALYs) and mean incremental effectiveness (7.18 ± 5.30 days free of wound) were favorable to punch grafting. All sensitivity analyses proved the robustness of our models. To conclude, punch grafting is the dominant alternative over usual care because it is cheaper and its utility and effectiveness are greater.

## 1. Introduction

Chronic wounds are a major health problem (silent epidemic), affecting millions of people in developed countries [1], although estimation of their true impact is difficult since the international consensus about when a wound should be considered chronic has not been reached [2,3]. The most common types of chronic wounds are pressure ulcers, diabetic foot ulcers, and leg ulcers of vascular etiology [1], and their prevalence is expected to rise with the aging of populations and the concurrent increase in predisposing diseases, such as diabetes, hypertension, and obesity [1,4]. In a recent systematic review of the literature, chronic wounds of various etiologies showed a pooled prevalence of 2.21 per 1000 population; among them, chronic leg ulcers were by far the most frequent type, with an estimated prevalence of 1.51 per 1000 population [2].

The true cost of treating a chronic wound worldwide is unknown due to a lack of both a clear definition and cross-country costs analyses, but available information strongly suggests that chronic wounds represent a large economic burden [2,3,5,6]. After conducting a systematic review of the literature, Chan et al. found a global mean cost of USD $23,300 ± 17,700 per patient (adjusted to 2015 USD), considering a follow-up period of one year and a public health care payer perspective [5]. Therefore, it is important to implement cost-effective techniques to heal chronic wounds.

Autologous skin grafting is a traditional technique used to promote epithelialization of hard-to-heal wounds resistant to adequate conventional treatment. The most-used type of thin split-thickness skin graft is the mesh graft obtained with dermatome; this technique requires sophisticated material and must be performed in the operating room with the patient under general or loco-regional anesthesia [7]. Punch grafting is another type of thin split-thickness skin graft, less aggressive and more simple than the mesh graft, which does not require the use of complex instruments [8,9,10] and can even be performed on an outpatient basis. Being carried out on an outpatient basis is interesting from an economic point of view, as it minimizes costs compared to hospital level implementation [11].

The literature reporting the clinical results of punch grafting on chronic wounds mainly consists of case series and case reports [8,10,11,12,13,14,15,16,17,18,19], but comparative studies are scarce. To the best of our knowledge, only one study compared punch grafting to negative wound therapy for the treatment of postsurgical wounds after melanoma excision [20], but comparative studies in the field of chronic wounds are lacking. The impact of punch grafting on patients’ quality of life has not been studied despite chronic wounds having a marked negative impact across all areas of quality of life of patients [21].

Concerning economic evaluations, a cost-minimization study compared punch grafting performed on an inpatient basis versus outpatient basis [11], but comparisons of punch grafting against usual care or other techniques are also lacking.

The main goal of this study was to conduct a cost-utility analysis (CUA) and a cost-effectiveness analysis (CEA) comparing punch grafting with usual care in an outpatient specialized wound clinic part of a dermatology department in the Spanish National Health system.

## 2. Materials and Methods

This study was conducted following the recommendations of the Consolidated Health Economic Evaluation Reporting Standards (CHEERS; Table S1) [22,23], aiming to improve the quality of health economic evaluations, as well as specific guidelines for retrospective studies [24] and for real-world data studies [25], which were published by the International Society for Pharmacoeconomics and Outcomes Research (ISPOR).

### 2.1. Background

#### 2.1.1. Flowchart and Decision Making

Patients are usually referred to our specialized wound clinic due to hard-to-heal wounds. Most patients are treated on an outpatient basis, although some of them are occasionally admitted to receive specific in-hospital treatment. To promote healing, ulcers are always cleansed and treated with sharp debridement as many times as considered necessary (Appendix A). By the time the research was conducted, ulcers presenting with a properly prepared wound bed but where epithelialization from the edges was clearly stagnant were treated with punch grafting (Appendix B) (this is no longer the situation in this specialized wound clinic, as the vast majority of ulcers are currently treated with punch grafting).

#### 2.1.2. Research “Wound-PRO-Spain”

The multi-center study “Wound-PRO-Spain” aimed to validate the wound-QoL questionnaire [26] for Spanish patients with wounds [27]. This study was conducted during the years 2016 and 2017, and the participating patients were asked to complete several health questionnaires, mainly EuroQoL-5D (EQ-5D) [28] and wound-QoL, at two specific time points, which were at baseline (baseline visit) and at three months (follow-up visit).

Notably, patients were treated according to the usual clinical practice carried out in each participating center, since the goal of that research (to validate a health questionnaire) was totally unrelated to the type of treatment provided. Therefore, the decision to perform a specific technique on each patient (in our case, punch grafting) and when to perform it, was not conditioned by the patient’s participation in the aforementioned “Wound-PRO-Spain” study.

### 2.2. Study Design

Retrospective observational study.

### 2.3. Setting and Location

A specialized wound clinic in a Spanish tertiary hospital.

### 2.4. Target Population

Eligible patients were patients with chronic wounds who participated in the “Wound-PRO-Spain” multi-center study.

The inclusion criteria were adult patients being treated exclusively at the wound clinic of Centro de Especialidades Vicente Soldevilla, which is part of the Hospital Universitario Infanta Leonor, Madrid.

The exclusion criteria were minors or patients who were treated in other centers different from the wound clinic mentioned above, patients who had already been treated with punch grafting before their participation in the “Wound-PRO-Spain” study, patients with wounds larger than 200 cm^2^, as these large wounds were not suitable for treatment with punch grafting in the wound clinic on the dates of the “Wound-PRO-Spain” study, and patients whose follow-up visit occurred beyond the three months foreseen in the design of the “Wound-PRO-Spain” study.

### 2.5. Study Groups/Comparators

For the purpose of this research, we included all patients who were treated with punch grafting at some point between the baseline visit and the follow-up visit in the “Wound-PRO-Spain” study in the PUNCH group. Therefore, the NoPUNCH group was composed of patients who did not receive punch grafting.

Some patients were treated with punch grafting after their participation in the “Wound-PRO-Spain” study; these patients were also assigned to the NoPUNCH group, as their health condition corresponded to those of the non-grafted patients.

### 2.6. Data Source

With the exception of health resources consumed, most information required for this research was previously collected for the purpose of the “Wound-PRO-Spain” study, including demographic data, characteristics of wounds, clinical outcome, and patient-reported outcome.

Patient-reported outcomes were assessed at baseline and at follow-up visits using EQ-5D and wound-QoL questionnaires.

The 3-level version of EQ-5D (EQ-5D-3L) [28] is a multidimensional generic questionnaire used to assess health-related quality of life (HRQoL) through 5 domains derived from 5 items, and it refers to the day the survey is filled. Items are scored from 1 (“no problem”) to 3 (“extreme problems”). This questionnaire also includes a global subjective health evaluation using a visual analogue scale (EQ-VAS) that ranges from 0 (“the worst health you can imagine”) to 100 (“the best health”). Using Spanish tariffs [29], the EQ-5D scores were subsequently transformed into utility scores (EQ-5D utility). Values for utilities can range from 0 (death) to 1 (full health).

Wound-QoL [26,30] is a short multidimensional specific questionnaire measuring HRQoL in patients suffering from chronic wounds, and it refers to the week before the survey is filled. It is composed of 17 items scored from 0 (“not at all”, which is “the best”) to 4 (“very much”, which is “the worst”). The wound-QoL global score is calculated as the arithmetic mean over all items.

Finally, and for the purpose of this specific research, the type and number of health resources consumed during the Wound-PRO-Spain study period were identified from clinical charts.

### 2.7. Study Perspective

The perspective of our analysis was that of the payer (public healthcare system); therefore, only direct medical costs were considered.

### 2.8. Time Horizon

The time horizon selected was three months, mainly because this was the planned period of time for the “Wound-PRO-Spain” study, but this is also a common time horizon in the setting of chronic wounds [31,32].

### 2.9. Choice and Measurement of Health Outcomes

The primary outcome measure for the CUA was quality-adjusted life years (QALYs) for a 3-month time horizon. The QALY is an index that includes effects in terms of both the patient’s quality of life (utility) and the period of time in such a health state [33]. Therefore, QALYs were calculated as the area under the curve defined between the utility score at the baseline visit and the utility score at the follow-up visit three months later, based on the assumption that utilities followed a linear course over time between these two time points. However, for many patients, we found that they had been discharged due to complete wound healing before the planned deadline for the “Wound-PRO-Spain” study; for convenience, those patients were surveyed during their discharge visit. For this subgroup of patients, QALYs were calculated as the addition of two areas. The first area between baseline and follow-up visits was calculated as described above. Then, based on the post hoc assumption that the utility score would remain unchanged from the follow-up visit to the foreseen three months, the area under the line defined by these two time points was also calculated and added to the first one.

To account for differences in baseline utilities between patients, the zero value to calculate all areas for every single patient was assumed to be the utility score at the baseline visit instead of the absolute zero value.

The primary outcome measure for CEA was wound-free period, expressed in days. This indicator was calculated as the difference between 90 days (3 months) and the number of days elapsed from baseline visit to complete wound healing.

The percentage of wounds completely healed during the study period and days spent to complete healing were considered as secondary indicators of effectiveness. Other secondary indicators were the changes in wound size, in pain intensity, and in scores of HRQoL (EQ-5D utility, EQ-VAS, and wound-QoL) from baseline to follow-up visit.

### 2.10. Estimating Health Resources Consumed and Related Costs

The direct medical cost per patient was calculated by multiplying the health resources consumed (measured in natural units) by its country-specific unit costs (expressed in 2016 Euros). Since the time horizon was three months, no discount rate was applied.

Disease-specific health resources consumed by each patient for the treatment of their chronic wounds during the three months of the study period were registered from medical records, specifically, the number of hospital admissions, if any, and the number of visits to the wound clinic; visits were categorized as “surgical visit” in the case where sharp debridement or punch grafting were performed or “standard visit” when such minor surgical procedures were not performed.

Unit costs were obtained from the officially published public health prices [34]. Sharp debridement and punch grafting were considered minor surgical procedures. Thus, the unit cost of surgical visits was assigned as the cost appearing under the heading E 03.1.1.2.3.2: “Minimally invasive diagnostic or therapeutic outpatient surgical procedure performed by a physician under local anesthesia or without anesthesia with or without conscious sedation, requiring little or short post-operative care”, whereas the unit cost of standard visits was assigned as the cost appearing under the heading E 03.1.1.2.1.2.: “Outpatient consultations”.

### 2.11. Assumptions

The unit cost of standard and surgical visits was assumed to include the cost of all materials (see Appendix A and Appendix B).

### 2.12. Cost-Utility and Cost-Effectiveness Analysis (CUA and CEA)

Our CUA and CEA were carried out in a Bayesian framework [35,36,37,38,39,40,41,42]. A multiple regression analysis was conducted to verify if the results in costs, utility, and effectiveness could be explained by the treatment performed or by other covariates [43,44,45,46,47,48,49,50]. To do that, *Costs*, *QALYs*, and *Wound-free period* were selected as dependent variables. Details about variable selection are provided in online supplementary material (Supplementary File S1).

Our final model included the following six covariates: time of duration of the wound at basal visit, expressed in months (*WoundDuration*); whether the wound was localized or not in lower limbs, expressed as a dichotomous variable (*WoundLeg;* yes = 1; no = 0); wound size, expressed in cm^2^ (*WoundSize*); EQ-5D at baseline, expressed in utility score (*EQ-5D*); wound-QoL at baseline, expressed in global score (*Wound-QoL*); and whether the wound was grafted or not, expressed as a dichotomous variable (*Treatment*; yes = PUNCH = 1; no = NoPUNCH = 0).

Thus, for patient *i*, the linear regression model to explain costs (*c*) and utility (*u*) measured by *QALYs* was as follows.

CUA-Model:

*ci* = β11 + β12 × *WoundDuration_i_* + β13 × *WoundLeg*_i_ + β14 × *WoundSize_i_* + β15 × *EQ*-5*D_i_* + β16 × *Wound-QoL_i_* + β17 × *Treatment*_*i* + ε1*i*_

*ui* = β21 + β22 × *WoundDuration_i_* + β23 × *WoundLeg*_i_ + β24 × *WoundSize_i_* + β25 × *EQ*-5*D_i_* + β26 × *Wound-QoL*_i_ + β27 × *Treatment*_*i* + ε2*i*_

The linear regression model to explain costs (*c*) and effectiveness (*e*) measured by *Wound-free period* was as follows:

CEA-Model:

*c_i_* = β_11_ + β_12_ × *WoundDuration_i_* + β_13_ × *WoundLeg*_i_ + β_14_ × *WoundSize_i_* + β_15_ × *EQ*-5*D_i_* + β_16_ × *Wound-QoL*_i_ + β_17_ × *Treatment*_*i* + ε1*i*_

*e_i_* = β_31_ + β_32_ × *WoundDuration_i_* + β_33_ × *WoundLeg*_i_ + β_34_ × *WoundSize_i_* + β_35_ × *EQ*-5*D_i_* + β_36_ × *Wound-QoL*_i_ + β_37_ × *Treatment*_*i* + ε3*i*_

Costs were asymmetrically distributed, thus cost values were log-transformed to conduct our Bayesian CUA and CEA. The parameter estimation was initiated by determining the likelihood function for both costs and effectiveness. A multivariate normal distribution for log-transformed total costs and for utility and effectiveness was assumed.

For a full Bayesian analysis, the prior distributions for the coefficients of the regression model should be defined. Non-informative proper priors were considered, which was a multivariate normal prior distribution with a mean vector of zeros and covariance matrix 10^5^ × Identity-matrix(7) for the vector of means, and a Wishart distribution with 2 degrees of freedom and covariance matrix Identity-matrix(2) were considered as the prior distribution for the variance-covariance matrix.

Concerning the regression model, the expected means for costs, utility, effectiveness, and for all the coefficients of the models were estimated from the posterior distributions, as well as their 95% Bayesian credible intervals. The posterior distribution of the coefficients was estimated using Markov chain Monte Carlo (MCMC) [51,52], which are simulation methods used to facilitate the development of full Bayesian analysis. A first burn-in sample with 10,000 simulations was calculated and then discarded. Then, 100,000 further simulations were run, from which the main statistics of the coefficients were calculated. All CUAs and CEAs were conducted using OpenBUGS. Supplementary File S2 contains the OpenBUGS codes for CUA-Model and CEA-Model.

Usually, β coefficients for the covariate *Treatment* can be interpreted as the incremental cost (β_17_), the incremental utility (β_27_), and the incremental effectiveness (β_37_). However, this interpretation is invalid when a variable is log-transformed because it was asymmetrically distributed, as was the case for our variable *Costs*. Therefore, incremental cost cannot be considered as the β coefficient value for *Treatment* (β_17_). Under these conditions, the costs ratio (costs of PUNCH divided by costs of NoPUNCH) is preferred over incremental cost (costs of PUNCH minus costs of NoPUNCH). The costs ratio can be obtained from the exponential transformation of the β coefficient associated with the covariate *Treatment*, namely exp (β_17_). Thus, the relative incremental cost attributed to punch grafting, the treatment under evaluation compared to usual care, can be expressed as (exp (β_17_) − 1) × 100.

The incremental cost-utility ratio (ICUR) was calculated as the ratio between incremental cost and incremental utility, and it is expressed as euros per QALY gained. The incremental cost-effectiveness ratio (ICER) was calculated as the ratio between incremental cost and incremental effectiveness, and it is expressed as euros per day free of wound. The ICUR and ICER are the main parameters used to make decisions after conducting CUAs and CEAs, respectively, but they are calculated only in the case where alternative treatment (punch grafting, in our case) is found to be more effective yet more costly, or less costly yet less effective, than control treatment.

Finally, two x–y scatterplots were built to graphically show the posterior costs ratio and incremental utility (cost-utility plane) and incremental effectiveness (cost-effectiveness plane). The probability of preference for punch grafting treatment is displayed as a function of the willingness to pay for increasing utility in a QALY (cost-utility acceptability curve) and for increasing effectiveness in a day free of wound (cost-effectiveness acceptability curve).

### 2.13. Sensitivity Analysis

To determine the model robustness, both one-way sensitivity analyses and extreme scenario analysis were conducted [53,54].

Two types of one-way sensitivity analysis for variables presenting with uncertainty were conducted. For the first one, values for variables measuring costs, utility, and effectiveness were changed by ±10% for every single patient to assess for uncertainty regarding the health resources consumed and the unit costs attributed to those resources (for *Costs* in CUA and CEA), for uncertainty regarding collection of EQ-5D data or later calculation of EQ-5D utilities (for *QALYs* in CUA), and for uncertainty regarding dates where wounds were considered completely healed (for *Wound-free period* in CEA). Details about how ±10% was selected are provided in online supplementary material (Supplementary File S1). Additionally, to assess for uncertainty associated with how QALYs were calculated, the base case CUA was also reanalyzed after using QALYs calculated using a different method, specifically as the difference between EQ-5D utility scores at follow-up visit minus baseline visit [33].

Lastly, an extreme scenario analysis was conducted considering the worst scenario for PUNCH: values for *Costs* were increased by 10% for PUNCH and reduced by 10% for NoPUNCH, while values for *QALYs* and *Wound-free period* were reduced by 10% for PUNCH and increased by 10% for NoPUNCH.

### 2.14. Sample Size

This specific study was partly based on information collected after conducting previous research to validate the wound-QoL questionnaire in a Spanish population of 115 patients, the “Wound-PRO-Spain” research, 88 of which attended our wound clinic. Thus, the final sample size was 80 patients.

### 2.15. Frequentist Statistical Analyses

Continuous variables are represented by mean ± SD, but comparisons between groups were performed using Student’s *t*-test or nonparametric Mann-Whitney U test, as appropriate according to the Shapiro-Wilks normality test results.

Categorical variables are presented as number of patients (percentage) and were compared using Chi-square or Fisher’s exact test.

Level of significance was preestablished at 0.05 [55]. All these exploratory statistical analyses were conducted using both SPSS Statistics Version 24 (IBM SPSS Statistics, Version 22.0, Armonk, NY, USA) and R-Project (R Foundation for Statistical Computing, Vienna, Austria).

### 2.16. Ethical Issues

The current research was conducted following the rules of the Declaration of Helsinki of 1975, revised in 2013, and it was approved by our institutional review board (Comité Ético de Investigación Clínica del Hospital General Universitario Gregorio Marañón, code “coste-utilidad microinjertos”, on 3 February, 2020). We were waived from the obligation of asking for individual permission based on the retrospective nature of the research and as the most sensitive information had been previously obtained as part of another study (Estudio español sobre resultados comunicados por el paciente con heridas; Wound PRO-Spain), which had also been approved by the same committee (Comité Ético de Investigación Clínica del Hospital General Universitario Gregorio Marañón, code “Wound-PRO”, on 27 July, 2016).

## 3. Results

A total of 115 patients were included in the “Wound-PRO-Spain” study, but only 88 of them were eligible for this specific research since they attended the Centro de Especialidades Vicente Soldevilla, which is part of the Dermatology Department of the Hospital Infanta Leonor in Madrid.

Four patients who had been already treated with punch grafting before the baseline visit to the “Wound-PRO-Spain” study were not included, as well as other four patients whose follow-up visit occurred later than the planned three months. Patients whose follow-up visit occurred before the third month due to complete wound healing were included, and it was assumed that their health status would remain stable from then until the third month.

Overall, 80 patients were included for analysis. The PUNCH group consisted of 46 patients treated with punch grafting at any time between baseline and follow-up visits, whereas the NoPUNCH group comprised 34 patients not treated with grafting during that period; three patients who were treated with punch grafting after the follow-up visit were included in the NoPUNCH group since they received usual care while participating in the “Wound-PRO-Spain” study.

### 3.1. Demographic Characteristics

The NoPUNCH group presented a significantly higher percentage of leg ulcers and ulcers of vascular etiology (Table 1).

### 3.2. Cost Analysis

No patient needed hospital admission. Thus, there were no hospital costs to be considered in any group. All costs were derived from visits to our wound clinic.

Total costs were significantly lower for the PUNCH group due to a significantly lower consumption of health resources, mainly due to a significantly lower number of surgical visits (Table 2).

### 3.3. Health Outcomes

Most wounds healed before the second time point and the percentage of complete healing was similar between PUNCH and NoPUNCH groups (87% vs. 85%, respectively; *p* = 0.831, Chi-square test). However, time to complete healing was significant shorter (*p* = 0.020; Mann-Whitney U test) in the PUNCH group (47.8 ± 23.7 days; *n* = 40) compared to the NoPUNCH group (62.5 ± 22.5 days; *n* = 29). Consequently, the wound-free period was significantly longer for the PUNCH than for the NoPUNCH group (36.7 ± 26.2 vs. 23.6 ± 22.9, *p* = 0.034, Mann-Whitney U test).

QALYs were higher for PUNCH than for NoPUNCH, although this difference did not reach statistical significance (0.0507 ± 0.0464 vs. 0.0387 ± 0.0448, respectively; *p* = 0.222, Mann-Whitney U test).

Compared to NoPUNCH, PUNCH showed better health outcomes concerning EQ-5D utility, EQ-5D VAS, and wound-QoL, although differences were not significant (Table 3).

Decisions to treat any patient with punch grafting were made according to routine practice. The mean waiting time for patients to be grafted was 19.4 ± 17.8 days after the baseline visit, and the majority of grafts were performed during the first two weeks (56.5%).

*Time-to-grafting*, in days, was post hoc selected as an explanatory variable to conduct, under a frequentist perspective, three exploratory univariate regression analyses. These analyses showed a significant inverse relationship with *Wound-free period* (β coefficient: −0.398; *p* < 0.001) and a direct significant relationship with *Costs* (β coefficient: 0.015; *p* < 0.001), but the relationship with *QALYs* was found to be not significant (β coefficient: −56.140; *p* = 0.331).

### 3.4. Cost-Utility Analysis (CUA)

The Bayesian cost-utility analysis for the base case showed that the PUNCH group was dominant over the NoPUNCH group as the PUNCH group resulted in lower costs and higher QALYs (Table 4).

Regarding the costs, ceteris paribus, punch grafting was associated with a relevant reduction of 37% in costs compared to usual care, as indicated by the mean value of the Costs-Ratio coefficient (0.63 ± 0.10) with a posterior 95% Bayesian credible interval of 0.48 to 0.80 (Table 4, CUA-Model, first row).

Concerning the utility, ceteris paribus, patients who were treated with punch grafting experienced an increase in utility of 0.02 ± 0.03 QALYs over patients who were only treated with usual care, as indicated by the value of the β_27_ coefficient in the CUA-Model, which usually allows for estimating the incremental utility; however, this increment was not relevant since its posterior 95% Bayesian credible interval was –0.03 to 0.06 (Table 4, CUA-Model, second row).

Regarding the regression model, the covariates *WoundLeg* and *Treatment* showed relevant explanatory power over *Costs*, indicating that treatment was more expensive for wounds located in lower limbs and that treating wounds with punch grafting was cheaper than usual care. No covariate showed relevant explanatory power over the QALYs (Table 5, CUA-Model).

**Figure 1:**
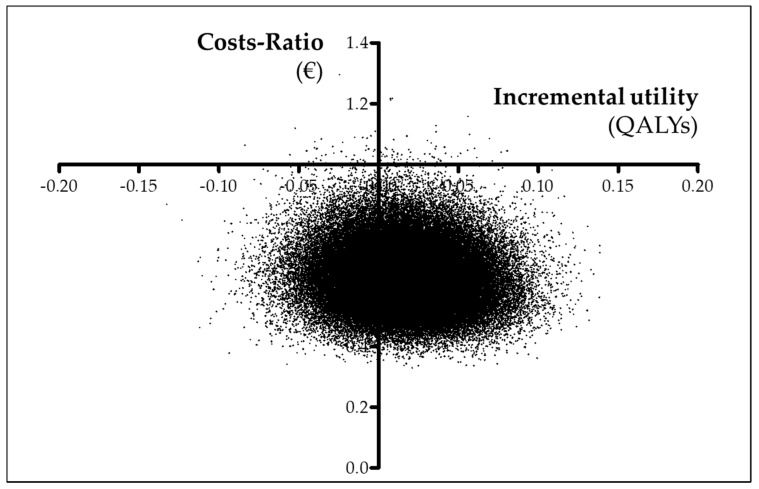
Cost-utility plane. Scatterplot showing the posterior costs ratio and incremental utility measured using QALYs.

**Figure 2:**
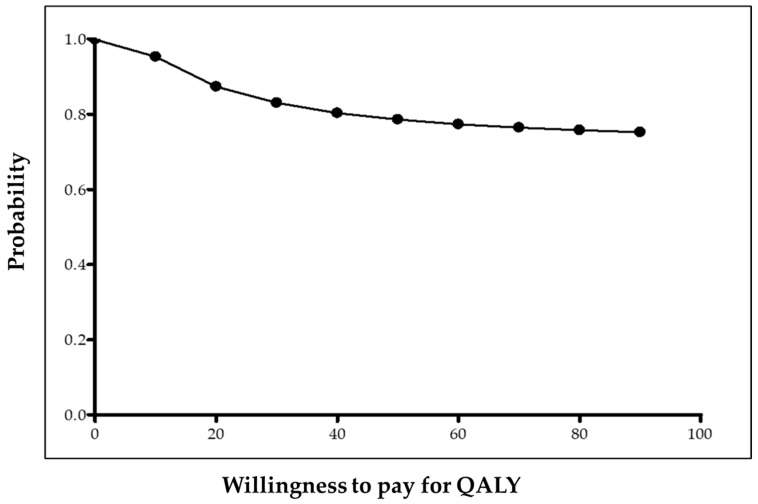
Cost-utility acceptability curve. The curve shows the cost–utility probabilities for PUNCH by different degrees of willingness to pay for QALYs.

Bayesian probability for PUNCH being cheaper was 99.89% and for PUNCH having more QALYs was 70.70%. Overall, the estimated probability for PUNCH being dominant was high (70.63%). Graphically, the preference for punch grafting over usual care is shown in the cost-utility plane (Figure 1) and in the cost-utility acceptability curve (Figure 2).

### 3.5. Cost-effectiveness analysis (CEA)

The Bayesian cost-effectiveness analysis for the base case also found PUNCH to be dominant. In addition to the aforementioned lower costs (Table 4, CEA-Model, first row), patients in this group were found to have more days free of wound (Table 4, CEA-Model, second row). Specifically, compared to usual care, ceteris paribus, wounds treated with punch grafting healed a week earlier (7.18 ± 5.30 days, as for β_37_ coefficient), yet this difference was not relevant (95% Bayesian interval: –1.57 to 15.89).

Logically, the influence of covariates over costs was similar for both the CEA-Model (Table 5) and CUA-Model. Regarding effectiveness, covariates *WoundLeg* and *WoundSize* showed relevant explanatory power over *Wound-free period* (Table 5, CEA-Model), indicating that patients suffering from wounds in lower limbs benefited from less days without wound and that the larger the wound size, the fewer the days free of wound.

**Figure 3:**
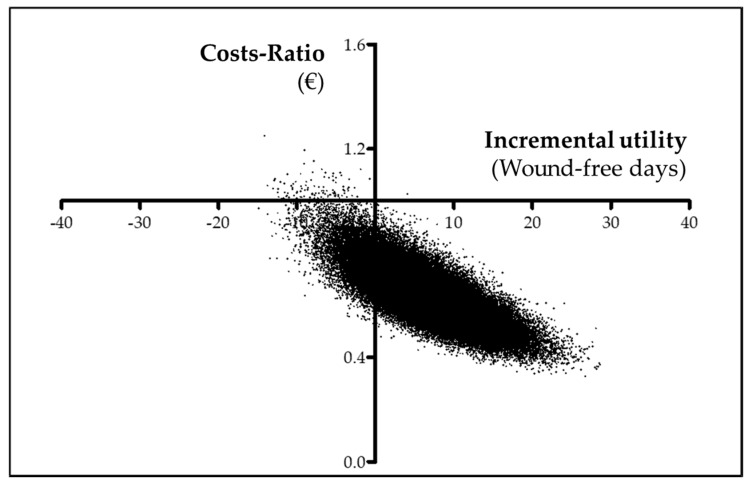
Cost-effectiveness plane. Scatterplot showing the posterior costs ratio and incremental effectiveness measured by days free of wound.

**Figure 4:**
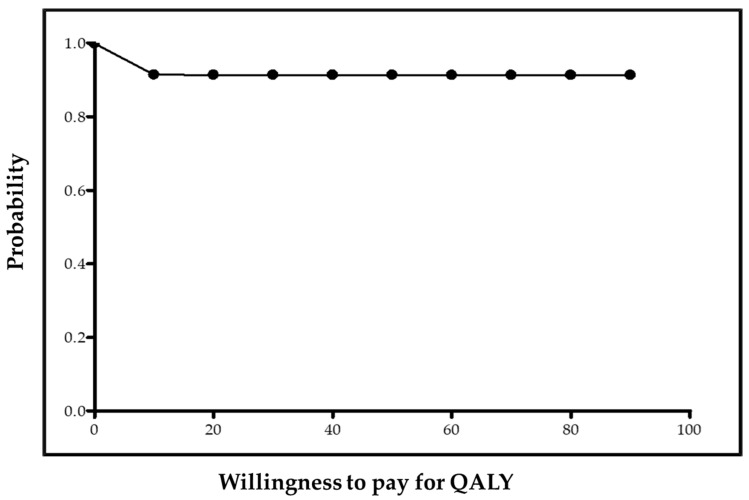
Cost-effectiveness acceptability curve. The curve shows the cost-effectiveness probabilities for PUNCH by different degrees of willingness to pay for wound-free day.

In addition to the previously shown 99.89% probability for PUNCH being cheaper, the Bayesian probability for PUNCH being more effective was 91.29%, and overall, the estimated probability for PUNCH being dominant was very high (91.28%). Graphically, the preference for punch grafting over usual care is shown in the cost-effectiveness plane (Figure 3) and in the cost-effectiveness acceptability curve (Figure 4).

### 3.6. Sensitivity Analysis

PUNCH was found to be the dominant alternative in all one-way sensitivity analyses conducted; among them, the lowest probability (68.86%) was found for a base case CUA reanalyzed after changing QALYs by –10%. Logically, extreme scenario analysis yielded the lowest probabilities for PUNCH to be dominant in both CUA (57.26%) and CEA (61.30%) (Supplementary Tables S2–S5).

Finally, two other types of sensitivity analyses were performed post hoc, which consisted of repeating the base case CUA and CEA for two subgroups.

The first subgroup was composed of 36 patients in the PUNCH group and 33 patients in the NoPUNCH group who suffered from wounds in lower limbs exclusively, as the variable *WoundLeg* was found to be significantly unevenly distributed among groups in the frequentist exploratory analysis (Table 1) and exhibited a Bayesian relevant influence on variables related to *Costs* and *Wound-free period* (Table 5). PUNCH was found to be the dominant alternative in the CUA (estimated probability of 68.51%) and in the CEA (estimated probability of 87.94%).

The second subgroup was composed of 26 pairs of patients who were matched between groups according to the size of their wounds, as the variable *WoundSize* was found to have a relevant influence on the variable *Wound-free period* (Table 5). Once again, PUNCH was found to be the dominant alternative in the CUA (estimated probability of 59.17%) and in the CEA (estimated probability of 76.47%).

## 4. Discussion

The main finding of the Bayesian CUA and CEA conducted was that punch was the dominant alternative over usual care for the treatment of outpatients suffering from chronic wounds, providing the patients were treated in a specialized wound clinic. Punch grafting was dominant due to being less expensive and having more utility and effectiveness compared with usual care. Expressed in probabilistic terms, the estimated probability found for punch grafting to be the dominant alternative was high in CUA (70.63%) and very high in CEA (91.28%).

Concerning CEA, the regression analyses showed that differences in costs and effectiveness were not only due to the treatment received but also to the characteristics of wounds.

Regarding effectiveness, both the less time needed to achieve complete healing and, consequently, the longer wound-free period found for PUNCH seemed to be dependent on the wound size (the larger the wound, the longer the time needed to heal) and on the location of the wound in lower limbs (longer healing times for wounds in lower limbs). The latter may be explained by the high prevalence of ulcers of vascular etiology and diabetic foot in lower limbs [56], as ulcers of these etiologies are usually hard-to-heal wounds.

The lower costs found for PUNCH seemed to be dependent on the location of the wound in lower limbs and on the treatment received; the latter makes sense and has two explanations. First, routine practice after grafting consists of conservative cleansing of the wounds; thus, the relative amount of surgical visits, which were the most expensive, was found to be significantly lower for the PUNCH group in the frequentist exploratory analysis. Second, as wounds treated with punch grafting healed sooner and patients in the PUNCH group were discharged earlier, the absolute amount of visits to the clinic was also significantly lower (Table 2).

Two post hoc sensitivity analyses were conducted to remove the eventual influence identified in Bayesian regression analyses of the wound characteristics—namely location in legs and wound size—on the outcomes. The base case CUA and CEA were reanalyzed for two subgroups: one composed of patients suffering only from leg ulcers and the other one of pairs of patients matched according to wound size. Remarkably, punch grafting was found to remain the dominant alternative after removing the influence of location and size.

Assuming punch grafting is the dominant alternative, its influence on effectiveness and utility was not the same. Incremental differences favored the PUNCH group (Table 4) but incremental effectiveness was noticeable (seven days free of wound), whereas incremental utility was almost negligible (0.02 QALYs). This small incremental utility could be considered a logical outcome as QALYs were calculated based on EQ-5D scores, and this questionnaire only considers the day the patient is surveyed. As the percentage of wounds completely healed at follow-up visit was similar between both study groups, patients could be expected to achieve similar scores in the EQ-5D survey regardless of the treatment they received. Generally, differences in QALYs would change in parallel with differences in healing rates between study groups [57]. Therefore, CEAs could be considered a better choice than CUAs to conduct an economic evaluation in the field of chronic wounds if the wound healing rate is expected to be very similar between groups [11].

As previously stated, punch grafting was found to be significantly less costly than usual care through shortening the healing time [58]. Thus, a direct relationship between costs and time to graft could be expected. Post hoc frequentist univariate regression analyses were conducted only for the PUNCH group to explore if any eventual relationship existed between time to grafting and costs, wound-free period, and QALYs. As expected, we found a direct and significant relationship between time to grafting and costs, and a logical inverse relationship with wound-free period. Notably, no significant relationship with QALYs was found, indicating that patients’ reported health outcomes were not influenced by the time of grafting, which reinforces the previous idea of CUAs being less indicated than CEAs. Given these results, punch grafting should be recommended over usual care, and this technique should be performed as soon as possible.

### 4.1. Comparison with Literature

Punch grafting has received little attention in the field of economic evaluations; to the best of our knowledge, only one study is available. Öien et al. [11] evaluated the costs of treating venous leg ulcers with punch grafting comparing two settings: hospital (inpatients) versus primary care setting (outpatients). Healing rate within 12 weeks was chosen as the main indicator of effectiveness. As both groups showed the same healing rate, 31%, the authors performed a cost-minimization analysis, and outpatient treatment was found to be cheaper. A direct comparison between this cost-minimization analysis and our CUA and CEA is precluded due to insurmountable differences in the type of economic evaluation and in the groups compared. Costs attributed to their group of outpatients could not be compared to costs attributed to our PUNCH group due to differences in subjects included for analysis. Specifically, in addition to the cost of visits to the clinic, they considered costs derived from travelling time, but we did not. The only relative similarity between our studies was reliance on the ratio between the unit cost attributed to a standard visit and those attributed to a surgical visit. Öien et al. assumed costs of 1997 £67 and 1997 £101, respectively (which would correspond to 2016 €144 and 2016 €218). Our unit costs for the same concepts were 2016 €71 for a standard visit and 2016 €145 for a surgical visit, which makes the ratio between unit costs of consults very similar to that of Öien et al. It suggests that the unit costs we attributed to each type of visit were reasonable.

### 4.2. Study Perspective

Instead of the societal perspective, we chose the public payer perspective to perform this research, as chosen by many other authors [31,32]. Later, all patients included in the study were found to be retired; this finding could have both favorable and unfavorable interpretations. This means that our evaluation of costs was not influenced by the perspective selected because indirect costs derived from loss of productiveness related to the societal perspective were not applicable [59]. However, this could compromise the external validity of the work, specifically the generalization of the results to not retired patients. However, this eventual shortfall would be of limited transcendence as indirect costs may have little impact on total costs. In a study conducted in Germany describing costs associated with chronic leg ulcers, indirect costs represented only 10% of total costs (direct and indirect) [60]. Societal perspective has rarely been applied to date; for instance, a recent review found that societal perspective was chosen in only 14% of CEAs conducted on chronic wounds [31].

### 4.3. Strengths

In our opinion, this work has several strengths that deserve further remark:

- Chronic wounds have been the subject of many CEAs comparing a variety of therapeutic alternatives [31,32], but as far as we know, no CEA comparison of punch grafting versus usual care or another technique had been conducted until now. In general, punch grafting is rarely investigated, and comparative studies of any kind were lacking until now, regardless of the type of wounds treated. Therefore, this research is the first CEA and CUA comparing punch grafting versus usual care.
- Wound healing is frequently selected as an indicator of effectiveness in many reported economic evaluations (44%), which is logical in the field of chronic wounds, whereas wound-free period and QALYs are less frequently selected as indicators (15% and 17%, respectively) [31,32]. In our case, using these two indicators to conduct our CEA and CUA, we simultaneously assessed the influence of punch grafting on healing time of the wounds as well as on the quality of life of patients.
- Regression models were built to explore whether differences in costs, wound-free period, and QALYs between both groups could be explained by other variables different to treatment. Such models allowed us to combine the advantages of incorporating patients’ characteristics through covariates in CUA and CEA, with the advantages of the Bayesian methodology. A remarkable advantage of incorporating covariates was that they allowed for differences between treatment groups in costs, utility, and effectiveness to be modelled as if they were completely homogeneous, except for the treatment received. They also allowed us to identify the part of the difference in costs, utility, and effectiveness that was not attributable to the treatment but to the differences in the characteristics of the patients. In addition, covariates allowed us to reduce the bias and uncertainty of the estimation of the coefficients [47,48,49,50]. Our regression model specification was properly evaluated as it fitted the checklist developed to assess statistical methods for addressing selection bias in CEAs based on observational data [61]. The selection of a Bayesian perspective instead of a standard frequentist one provided two advantages. First, it allowed for the incorporation of a priori information through a dynamic process. Second, it allowed for the results of CUAs and CEAs to be interpreted in terms of probability. More specifically, the cost-utility and cost-effectiveness acceptability curves were able to be interpreted in terms of probability; as stated before, such interpretation is only possible when a Bayesian perspective is adopted [49].
- We conducted a retrospective observational study based on patients who had been treated by their attending physicians according to their best clinical judgement. This kind of observational study based on individual patient data is thought to be more valid than clinical trials to generate real-world data, which, in turn, are thought to provide more of an advantage than a limitation when generalizing the results of specific research to day-to-day practice [62,63]. In addition, working with individual patient data allows real clinical outcomes of treatment to be captured [62]. In our opinion, this realistic approach to the assessment of treatment and outcomes represents an advantage over studies that try to model the natural history of the disease based on population data, as do Markov models, for instance [64].

### 4.4. Limitations

The main limitation of this observational retrospective study is that our patients were not randomly distributed to treatments with or without punch grafting, but they were treated according to clinical criteria. Thus, sample bias due to any specific characteristic of patients or wounds could not be completely ruled out. A first step to overcome any eventual bias and to reduce uncertainty was the development of a regression model. Variables that could affect cost, utility, and effectiveness were considered to specify the model to ascertain whether the treatments under evaluation had a real influence over the results of CUA and CEA. Our Bayesian regression model revealed that differences in the number of days free of wound, which was the main indicator of effectiveness in CEA, could not be explained by the treatments alone, but also by the size and the location of wounds in legs. Therefore, the base-case CUA and CEA were repeated for two subgroups: for those 69 patients suffering exclusively from leg ulcers and for 26 pairs of patients matched according to their wound size. Punch grafting remained the dominant alternative after conducting these sensitivity analyses (Supplementary Tables S2–S5). Considering all these analyses, we do not think our results were influenced by any sample bias, even accepting that sample bias was present.

This was a Spanish single-center study conducted in a specialized wound clinic. This specificity could affect the generalization of our findings to other less specialized settings in which punch grafting could eventually be either a more time- or health-resources-consuming technique (more costly) or less effective. Generalization to other countries should also be performed with caution since unit costs could be different in other socio-economic contexts. For instance, unit costs attributed to standard and surgical visits were lower in our case than reported by Öien et al. [11], even though the unit costs ratio between standard and surgical visits was found to be similar between both works. Nevertheless, punch grafting remained the dominant technique after conducting one-way sensitivity analysis, in which its costs were changed by ±10% and its utility and effectiveness changed by ±10%. Punch grafting remained dominant even under extreme scenario analysis, which was the worst scenario for PUNCH. Therefore, punch grafting can be considered dominant to usual care in terms of cost utility and cost effectiveness with a high degree of confidence [54].

Finally, the sample size was not calculated in anticipation. Our research was specifically planned to take advantage of information about HRQoL reported by patients who had participated in another previous and unrelated study (Spain-Wound-PRO) and, as stated before, who were treated in a single wound clinic. Thus, our final sample size of 80 patients was mainly restricted by these two conditions. Even so, many other studies analyzing different issues with chronic wounds had similar sample sizes [31].

## 5. Conclusions

The main findings of these Bayesian cost-utility and cost-effectiveness analyses comparing punch grafting versus usual care for the treatment of chronic wounds favored punch grafting, providing patients were treated in a specialized wound clinic on an outpatient basis. Punch grafting was found to be cheaper than usual care, and incremental differences for utility (QALYs) and effectiveness (wound-free period) also favored punch grafting, although these two latter differences were not relevant. Thus, punch grafting is the dominant alternative. In addition, time to grafting showed a significant direct relationship with costs and a significant inverse relationship with wound-free period. Taken altogether, our findings strongly suggest that punch-grafting should be recommended over usual care for the early treatment of chronic wounds. Ideally, a multicentric randomized clinical trial should be conducted to confirm our results.

## Figures and Tables

**Table 1:** Baseline characteristics of patients and wounds.

		PUNCH *n* = 46	NoPUNCH *n* = 34	*p*-Value
Patients	Age (years)	68.4 (16.9)	72.8 (12.9)	0.447 ^a^
	Sex, Female (%)	56.5	71.5	0.118 ^b^
	Diabetes (%)	32.6	44.1	0.293 ^b^
	Hypertension (%)	69.6	88.2	0.048 ^b^
	Smoker (%)	13.0	11.8	1.000 ^c^
	Pain caused by ulcers (NRS)	5.6 (3.2)	5.7 (3.5)	0.864 ^a^
Wounds	Etiology, vascular (%)	39.1	76.5	0.001 ^b^
	Location, legs (%)	78.3	97.1	0.020 ^c^
	Duration (months)	9.2 (10.7)	8.4 (10.3)	0.584 ^a^
	Size (cm^2^)	9.81 (14.98)	14.07 (22.82)	0.248 ^a^
	Deepness, limited to dermis (%)	76.1	67.7	0.503 ^b^

Data for quantitative variables are expressed as mean (SD). NRS, numerical rating scale; ^a^ Mann–Whitney U test; ^b^ Chi-square test; ^c^ Fisher exact test.

**Table 2:** Health resources, unit costs, and health costs.

	Health Resources (*n*)			Unit Costs (€)	Costs (€)		
	PUNCH (*n* = 46)	NoPUNCH (*n* = 34)	*p*-Value		PUNCH (*n* = 46)	NoPUNCH (*n* = 34)	*p*-Value
Surgical visit	3.5 (3.1)	9.9 (5.7)	<0.001	145	501.54 (455.16)	1431.41 (817.67)	<0.001
Standard visit	7.7 (6.4)	4.7 (2.1)	0.080	71	538.41 (446.09)	327.41 (146.72)	0.127
Total	11.2 (7.7)	14.6 (7.3)	0.031	---	1040 (705)	1759 (930)	<0.001

Data are expressed as mean (SD). Mann–Whitney U test for all comparisons.

**Table 3:** Comparison of change from baseline in HRQoL and ulcer pain and size.

	PUNCH *n* = 46			No PUNCH *n* = 34			*p*-Value for Differences
	Baseline	Follow Up	Difference	Baseline	Follow Up	Difference	
EQ-5D utility score	0.5494 (0.2571)	0.8383 (0.2528)	0.2889 (0.2716)	0.4633 (0.2463)	0.7138 (0.2526)	0.2505 (0.2876)	0.368
EQ-5D VAS score	63.37 (24.81)	81.09 (17.06)	17.72 (14.49)	61.15 (21.00)	77.50 (12.81)	16.35 (18.64)	0.777
Wound-QoL score	2.05 (0.96)	0.52 (0.64)	−1.53 (1.07)	2.30 (1.20)	0.98 (1.12)	−1.32 (1.50)	0.129
Ulcer pain (NRS)	5.6 (3.2)	0.7 (1.4)	−4.9 (3.4)	5.7 (3.5)	0.7 (1.4)	−5.0 (3.8)	0.864
Ulcer size (cm^2^)	9.81 (14.98)	0.18 (0.58)	−9.64 (14.94)	14.07 (22.81)	2.09 (10.36)	−11.98 (17.50)	0.275

Data are expressed as mean (SD). HRQoL, health-related quality of life; EQ-5D, generic questionnaire used to assess HRQoL; VAS, visual analogic scale; Wound-QoL, specific questionnaire measuring HRQoL in patients suffering from chronic wounds; NRS, numerical rating scale. Mann-Whitney *U* test for all comparisons.

**Table 4:** Statistical summary of costs, utility, and effectiveness (100,000 simulations MCMC).

	PUNCH	NoPUNCH	Incremental Difference
**CUA-Model**			
Costs (€)	1074 ± 112.2 (906.8; 1271.0)	1737 ± 209.4 (1426.0; 2105.0)	0.63 ± 0.10 **(0.48; 0.80)**
Utility (*QALYs*)	0.0523 ± 0.0187 (0.0218; 0.0830)	0.0364 ± 0.0220 (0.0002; 0.0724)	0.02 ± 0.03 (−0.03; 0.06)
**CEA-Model**			
Costs (€)	1075 ± 111.7 (908.1; 1271.0)	1737.0 ± 207.7 (1428.0; 2102.0)	0.63 ± 0.10 **(0.48; 0.80)**
Effectiveness (*Wound-free period*)	34.16 ± 3.35 (28.65; 39.63)	26.98 ± 3.93 (20.53; 33.42)	7.18 ± 5.30 (−1.57; 15.89)

Data expressed as mean ± SD (95% credible interval). MCMC, Markov chain Monte Carlo; QALYs, quality-adjusted life years; bold, intervals not including the zero value.

**Table 5:** Estimations of the posterior distribution of the β coefficients for the cost-utility and cost-effectiveness analysis (100,000 simulations MCMC).

		Mean (SD)	95% CI
**CUA-Model (DIC = 4.695)**			
Costs	β_11_ intercept	6.40 (0.35)	(5.82; 6.97)
	β_12_ WoundDuration	0.01 (0.01)	(−0.01; 0.02)
	β_13_ WoundLeg	0.56 (0.23)	(0.19; 0.92)
	β_14_ WoundSize	0.01 (0.00)	(−0.00; 0.01)
	β_15_ EQ-5D	0.12 (0.31)	(−0.39; 0.64)
	β_16_ Wound-QoL	0.09 (0.07)	(−0.03; 0.21)
	β_17_ Treatment	−0.48 (0.15)	(−0.73; –0.22)
	Costs-Ratio (exp β_17_)	0.63 (0.10)	(0.48; 0.80)
Utility	β_21_ intercept	0.09 (0.07)	(−0.02; 0.20)
	β_22_ WoundDuration	−0.00 (0.00)	(−0.00; 0.00)
	β_23_ WoundLeg	−0.03 (0.04)	(−0.10; 0.04)
	β_24_ WoundSize	−0.00 (0.00)	(−0.00; 0.00)
	β_25_ EQ-5D	−0.08 (0.06)	(−0.18; 0.02)
	β_26_ Wound-QoL	0.01 (0.01)	(−0.02; 0.03)
	β_27_ Treatment	0.02 (0.03)	(−0.03; 0.06)
**CEA-Model (DIC = 810.2)**			
Costs	β_11_ intercept	6.40 (0.35)	(5.82; 6.97)
	β_12_ WoundDuration	0.01 (0.01)	(−0.01; 0.02)
	β_13_ WoundLeg	0.56 (0.23)	(0.19; 0.92)
	β_14_ WoundSize	0.01 (0.00)	(−0.00; 0.01)
	β_15_ EQ-5D	0.12 (0.31)	(−0.39; 0.64)
	β_16_ Wound-QoL	0.09 (0.07)	(−0.03; 0.21)
	β_17_ Treatment	−0.48 (0.15)	(−0.73; –0.22)
	Costs-Ratio (exp β_17_)	0.63 (0.10)	(0.48; 0.80)
Effectiveness	β_31_ intercept	57.54 (12.25)	(37.41; 77.62)
	β_32_ WoundDuration	−0.39 (0.25)	(−0.80; 0.02)
	Β_33_ WoundLeg	−18.66 (7.86)	(−31.56; –5.76)
	β_34_ WoundSize	−0.35 (0.14)	(−0.57; –0.12)
	β_35_ EQ-5D	3.44 (10.91)	(−14.49; 21.33)
	β_36_ Wound-QoL	−4.06 (2.60)	(−8.35; 0.23)
	β_37_ Treatment	7.18 (5.30)	(−1.57; 15.89)

DIC, deviance information criterion; EQ-5D, generic questionnaire used to assess HRQoL; Wound-QoL, specific questionnaire measuring HRQoL in patients suffering from chronic wounds; CI, credible interval. Bold, intervals not including the zero value.

## Data Availability

Datasets used and/or analyzed during the current study are available from the corresponding author upon reasonable request.

## Supplementary Materials

The following are available online at https://www.mdpi.com/1660-4601/17/11/3823/s1, Table S1: Consolidated Health Economic Evaluation Reporting Standards (CHEERS) checklist: Items to include when reporting economic evaluations of health interventions, File S1: Frequentist exploratory analysis, File 2: OpenBUGS codes for CUA-Model and CEA-Model, Table S2: Sensitivity analysis of CUA-Model: Statistical summary of costs and utility (100,000 simulations MCMC), Table S3: Sensitivity analysis of CUA-Model: Estimations of the posterior distribution of the β coefficients and the probabilities related to the cost-utility analysis (100,000 simulations MCMC), Table S4: Sensitivity analysis of CEA-Model: Statistical summary of costs and effectiveness (100,000 simulations MCMC), Table S5: Sensitivity analysis of CEA-Model: Estimations of the posterior distribution of the β coefficients and of the probabilities related to the cost-effectiveness analysis (100,000 simulations MCMC).

## Appendix A. Sharp Debridement

Debridement has a significant role to play in the wound healing process, as it helps to remove devitalized tissue from the wound bed. Different types of debridement exist; however, the debridement technique commonly used in our clinic is sharp debridement, as it is considered the most efficient method to promote the granulation phase of wound healing [65,66].

To achieve adequate analgesia for debridement, we use the anesthetic lidocaine-prilocaine cream (EMLA). The procedure of debridement is always preceded by wound cleansing with tap water or saline. Afterwards EMLA cream is applied for 30–40 min, in occlusion with a plastic film. Sharp debridement consists of removal of dead tissue with scalpel or curette and tweezers often just above the level of viable tissue, so it can be considered a minor surgical procedure. It is a quick and selective procedure in experienced hands, so it should be carried out by a practitioner with the proven skills and knowledge [65,66].

When the wound bed does not present with sloughy tissue that needs to be removed, cleansing is enough before dressing changes and bandage application. Moreover, those wounds that have been grafted should never be debrided in the follow-up visits, and cleansing should be slight not to remove cells and growth factors that will help the healing process.

## Appendix B. Punch Grafting

Different methods are used to obtain the punch grafts, since a curette, scissors, scalpel, or punch can be used, but the anatomical knowledge of the skin and the experience of the specialist are key to systematically and quickly performing the procedure. These grafts are obtained after local anesthesia with 2% lidocaine in the donor site, which is usually the thigh. The skin fragments usually have a diameter of between 4 and 6 mm and, in terms of depth, do not go deeper than the papillary dermis, which is identified by point-sized bleeding. The grafts obtained from the donor site are placed with tweezers on the wound bed, separated by a few millimeters, and the already-grafted wound is normally covered with a primary non-adherent dressing, a secondary dressing, and compression bandage or discharge, depending on the etiology. To promote graft taking, pressure and local immobilization must be maintained during the first days. However, graft taking also depends on other factors, such as the characteristics of the wound bed (granulation tissue, slough, and bacterial load) [10]. This technique is performed on an outpatient basis, but it is sometimes necessary to admit the patient to ensure immobilization compliance.

Conservative cleansing is essential in the first dressing changes after punch grafting, avoiding any kind of debridement to avoid detaching the micrografts and to maintain the microenvironment that has been created. The first dressing is usually changed after approximately 5 days and usually consists of careful washing with saline. Then, the same procedure is performed once or twice per week until complete healing.

Sometimes not all the grafts attach, but an advantage of this technique is that it can be repeated as many times as necessary until complete epithelization is achieved. In our clinical practice, we have confirmed that even if the wound bed does not present with perfect conditions for grafting, punch grafts that do not successfully adhere release growth factors and cells that promote wound epithelialization and reduce pain. Punch grafting in painful ulcers may produce a striking early analgesic effect [8,9,10] and is therefore a technique that is very well accepted by patients.

The donor site heals by secondary intention, with minimal infection or bleeding risks. Alginate is commonly recommended as primary dressing to minimize the risk of bleeding in the first 48–72 h. In the long term, the donor area may present color changes, both hypo and hyperpigmentation [10].
