# Supplementary material for: Bayesian Regression Model for a Cost-Utility and Cost-Effectiveness Analysis Comparing Punch Grafting Versus Usual Care for the Treatment of Chronic Wounds
Source: Int J Environ Res Public Health. 2020 May 28;17(11):3823. doi: 10.3390/ijerph17113823 (PMC7313055; doi:10.3390/ijerph17113823)
Supplement: Supplementary file 1 [file ijerph-17-03823-s001.zip › Supplementary File S1. Frequentist exploratory analysis.docx]

**Supplementary File S1. Frequentist exploratory analysis.**

**Selection of covariates for Bayesian regression model.**

Multiple linear regression analysis was performed to examine the relationship between potential predictive factors and dependent variables (costs, utility, and effectiveness). Covariates to be included in a linear regression model were selected after a three-step process. First, level of association between all baseline variables (age, gender, diabetes mellitus, hypertension, smoker, analgesics, wound size, wound etiology, wound location, wound deepness, wound duration, wound pain, EQ-5D utility, wound-QoL score) was explored using Spearman correlation test or Chi-square test, as appropriate. Second, the eventual relationship between each dependent variable and all independent basal variables was explored through a simple linear regression, and those covariates reaching a level of significance less than 0.2 for at least two dependent variables were chosen for the exploratory multivariate analysis. Third, these covariates were included in the multivariate regression model, using the backward elimination method. Finally, covariates for the definitive multiple regression analysis were selected according to both statistical and clinical criteria.

**Selection of a variation of ±10% to conduct sensitivity analysis.**

Actual means and 95% confidence intervals for the three dependent variables (Cost, QALYs, Wound-free period) were highly influenced by extreme values because of the relatively small sample size. Thus, bootstrapping technique (1000 runs, default value in SPSS) was used to obtain more steady means and confidence intervals. Stratified bootstrapping was performed in the following way: Cost was stratified by QALYs and Wound-free period, while QALYs and Wound-free period were stratified by Cost.

The percentages of variation for the lower and upper limits of the steady 95%CI were lower than 10% (≈3%, ≈8% and ≈3%, respectively). In order to conduct sensitivity analysis under stricter conditions to improve generalization of the results to different contexts, we decided to select a 10% instead of these percentages of variation.
