# Supplementary material for: Bayesian Regression Model for a Cost-Utility and Cost-Effectiveness Analysis Comparing Punch Grafting Versus Usual Care for the Treatment of Chronic Wounds
Source: Int J Environ Res Public Health. 2020 May 28;17(11):3823. doi: 10.3390/ijerph17113823 (PMC7313055; doi:10.3390/ijerph17113823)
Supplement: Supplementary file 1 [file ijerph-17-03823-s001.zip › Supplementary File S2 OpenBUGS codes for CUA-Model and CEA-Model NUEVO.docx]

**Supplementary File S2.** OpenBUGS codes for CUA-Model and CEA-Model.

#**CUA-Model**{

for(i in 1:N){

output[i,1] <- utility[i]

output[i,2] <- log(cost[i])

output[i,1:2] ~ dmnorm(mu[i,1:2],v[1:2,1:2])

mu[i,1] <- beta1[1] + beta1[2]*WoundDuration[i] + beta1[3]*WoundLeg[i]+beta1[4]*WoundSize[i] + beta1[5]*EQ5D[i] + beta1[6]*WoundQoL[i]+beta1[7]*Treatment[i]

mu[i,2] <- beta2[1] + beta2[2]*WoundDuration[i] + beta2[3]*WounLocation[i]+beta2[4]*WoundSize[i] + beta2[5]*EQ5D[i] + beta2[6]*WoundQoL[i]+beta2[7]*Treatment[i]}

beta1[1:7] ~ dmnorm(a[], A[,])

beta2[1:7] ~ dmnorm(b[], B[,])

v[1:2,1:2] ~ dwish(A1[,],f1)

#Mean utility.

UtilityMeanT1 <- beta1[1] + beta1[2]*8.8875 + beta1[3]*0.8625+beta1[4]*11.6223 + beta1[5]*0.5128 + beta1[6]*2.1540+ beta1[7]

UtilityMeanT0 <-beta1[1] + beta1[2]*8.8875 + beta1[3]*0.8625 + beta1[4]*11.6223 + beta1[5]*0.5128 + beta1[6]*2.1540

#Mean cost.

Var[1:2,1:2]<-inverse(v[1:2,1:2])

mumedioT1<-beta2[1] + beta2[2]*8.8875 + beta2[3]*0.8625+beta2[4]*11.6223 + beta2[5]*0.5128 + beta2[6]*2.1540 + beta2[7]

MeanCostT1 <- exp(mumedioT1+Var[2,2]/2)

mumedioT0<-beta2[1] + beta2[2]*8.8875 + beta2[3]*0.8625+beta2[4]*11.6223 + beta2[5]*0.5128 + beta2[6]*2.1540

MeanCostT0 <- exp(mumedioT0+Var[2,2]/2)

# Incremental utility and incremental cost.

DeltaE <- beta1[7]

ratioC <- exp(beta2[7])

# Cost-utility acceptability curve.

for(k in 1:NK){

Q[k] <- step(Rc[k]*DeltaE - (ratioC-1))}

# Probabilities.

effec <- step(beta1[7])

cheap <- 1-step(exp(beta2[7])-1)

dominant <- effec*cheap}

# Priors (non-informative).

list(a=c(0,0,0,0,0,0,0), A=structure(.Data = c(0.00001,0,0,0,0,0,0,0,0.00001,0,0,0,0,0,0,0,0.00001,0,0,0,0,0,0,0,0.00001,0,0,0,0,0,0,0,0.00001,0,0,0,0,0,0,0,0.00001,0,0,0,0,0,0,0,0.00001),.Dim=c(7,7)),

b=c(0,0,0,0,0,0,0), B=structure(.Data = c(0.00001,0,0,0,0,0,0,0,0.00001,0,0,0,0,0,0,0,0.00001,0,0,0,0,0,0,0,0.00001,0,0,0,0,0,0,0,0.00001,0,0,0,0,0,0,0,0.00001,0,0,0,0,0,0,0,0.00001), .Dim=c(7,7)), A1=structure(.Data =

c(1,0,0,1),.Dim=c(2,2)), f1=2)

#Data.

list(N=80)

Treatment[]cost[]utility[]WoundDuration[]WoundLocation[]WoundSize[]EQ5D[]WoundQoL[]

1 935 0.0715 4 1 0.06 0.493 2.94

1 2026 -0.0195 18 1 1.77 0.6839 1.65

(…76 more rows)

0 3262 0.0459 3 1 16.49 0.2215 3.59

0 1062 0.0783 7 1 1.18 0.3227 2.65

END

# Rc values.

list(NK=10, Rc=c(0,10,20,30,40,50,60,70,80,90))

# Starting values.

list(v=structure(.Data=c(1,0,0,1),.Dim=c(2,2)), beta1=c(1,1,1,1,1,1,1), beta2=c(1,1,1,1,1,1,1))

#**CEA-Model**{

for(i in 1:N){

output[i,1] <- effectiveness[i]

output[i,2] <- log(cost[i])

output[i,1:2] ~ dmnorm(mu[i,1:2],v[1:2,1:2])

mu[i,1] <- beta1[1] + beta1[2]*WoundDuration[i] + beta1[3]*WoundLeg[i]+beta1[4]*WoundSize[i] + beta1[5]*EQ5D[i] + beta1[6]*WoundQoL[i]+beta1[7]*Treatment[i]

mu[i,2] <- beta2[1] + beta2[2]*WoundDuration[i] + beta2[3]*WounLocation[i]+beta2[4]*WoundSize[i] + beta2[5]*EQ5D[i] + beta2[6]*WoundQoL[i]+beta2[7]*Treatment[i]}

beta1[1:7] ~ dmnorm(a[], A[,])

beta2[1:7] ~ dmnorm(b[], B[,])

v[1:2,1:2] ~ dwish(A1[,],f1)

#Mean effectiveness.

EffectivenessMeanT1 <- beta1[1] + beta1[2]*8.8875 + beta1[3]*0.8625+beta1[4]*11.6223 + beta1[5]*0.5128 + beta1[6]*2.1540+ beta1[7]

EffectivenessMeanT0 <-beta1[1] + beta1[2]*8.8875 + beta1[3]*0.8625 + beta1[4]*11.6223 + beta1[5]*0.5128 + beta1[6]*2.1540

#Mean cost.

Var[1:2,1:2]<-inverse(v[1:2,1:2])

mumedioT1<-beta2[1] + beta2[2]*8.8875 + beta2[3]*0.8625+beta2[4]*11.6223 + beta2[5]*0.5128 + beta2[6]*2.1540 + beta2[7]

MeanCostT1 <- exp(mumedioT1+Var[2,2]/2)

mumedioT0<-beta2[1] + beta2[2]*8.8875 + beta2[3]*0.8625+beta2[4]*11.6223 + beta2[5]*0.5128 + beta2[6]*2.1540

MeanCostT0 <- exp(mumedioT0+Var[2,2]/2)

# Incremental effectiveness and incremental cost.

DeltaE <- beta1[7]

ratioC <- exp(beta2[7])

# Cost-effectiveness acceptability curve.

for (k in 1:NK){

Q[k] <- step(Rc[k]*DeltaE - (ratioC-1))}

# Probabilities.

effec <- step(beta1[7])

cheap <- 1-step(exp(beta2[7])-1)

dominant <- effec*cheap}

# Priors (non-informative).

list(a=c(0,0,0,0,0,0,0), A=structure(.Data = c(0.00001,0,0,0,0,0,0,0,0.00001,0,0,0,0,0,0,0,0.00001,0,0,0,0,0,0,0,0.00001,0,0,0,0,0,0,0,0.00001,0,0,0,0,0,0,0,0.00001,0,0,0,0,0,0,0,0.00001),.Dim=c(7,7)),

b=c(0,0,0,0,0,0,0), B=structure(.Data = c(0.00001,0,0,0,0,0,0,0,0.00001,0,0,0,0,0,0,0,0.00001,0,0,0,0,0,0,0,0.00001,0,0,0,0,0,0,0,0.00001,0,0,0,0,0,0,0,0.00001,0,0,0,0,0,0,0,0.00001), .Dim=c(7,7)), A1=structure(.Data =

c(1,0,0,1),.Dim=c(2,2)), f1=2)

#Data.

list(N=80)

Treatment[]cost[]effectiveness[]WoundDuration[]WoundLocation[]WoundSize[]EQ5D[]WoundQoL[]

1 935 27 4 1 0.06 0.493 2.94

1 2026 12 18 1 1.77 0.6839 1.65

(…76 more rows)

0 3262 0 3 1 16.49 0.2215 3.59

0 1062 58 7 1 1.18 0.3227 2.65

END

# Rc values.

list(NK=10, Rc=c(0,10,20,30,40,50,60,70,80,90))

# Starting values.

list(v=structure(.Data=c(1,0,0,1),.Dim=c(2,2)), beta1=c(1,1,1,1,1,1,1), beta2=c(1,1,1,1,1,1,1))
