# Supplementary material for: Bayesian Regression Model for a Cost-Utility and Cost-Effectiveness Analysis Comparing Punch Grafting Versus Usual Care for the Treatment of Chronic Wounds
Source: Int J Environ Res Public Health. 2020 May 28;17(11):3823. doi: 10.3390/ijerph17113823 (PMC7313055; doi:10.3390/ijerph17113823)
Supplement: Supplementary file 1 [file ijerph-17-03823-s001.zip › Supplementary Table S1. Checklist CHEERS.docx]

**Supplementary Table S1.** CHEERS checklist—Items to include when reporting economic evaluations of health interventions.

| **Section/ítem** | **Item**  **No** | **Recommendation** | **Reported on page No/line No** |
| --- | --- | --- | --- |
| **Title and abstract** |  |  |  |
| Title | 1 | Identify the study as an economic evaluation or use more specific terms such as “cost-effectiveness analysis”, and describe the interventions compared. | page 1, line 2 to 4 |
| Abstract | 2 | Provide a structured summary of objectives, perspective, setting, methods (including study design and inputs), results (including base case and  uncertainty analyses), and conclusions. | page 1, line 15 to 29 |
| **Introduction** |  |  |  |
| Background and  objectives | 3 | Provide an explicit statement of the broader context for the study. | page 1, line 34 to 42  page 2, line 58 to 67 |
|  |  | Present the study question and its relevance for health policy or practice decisions. | page 1, line 43 to 48  page 2, line 55 to 57  page 2, line 62 to 64  page 2, line 68 to 70 |
| **Methods** |  |  |  |
| Target population and subgroups | 4 | Describe characteristics of the base case population and subgroups analysed, including why they were chosen. | page 3, line 103 to 113  page 13, line 411 to 412 |
| Setting and location | 5 | State relevant aspects of the system(s) in which the decision(s) need(s) to be made. | page 3, line 101 to 102 |
| Study perspective | 6 | Describe the perspective of the study and relate this to the costs being evaluated. | page 4, line 141 to 143 |
| Comparators | 7 | Describe the interventions or strategies being compared and state why they were chosen. | page 3, line 114 to 121 |
| Time horizon | 8 | State the time horizon(s) over which costs and consequences are being evaluated and say why appropriate. | page 4, line 144 to 147 |
| Discount rate | 9 | Report the choice of discount rate(s) used for costs and outcomes and say why appropriate. | not applicable |
| Choice of health outcomes | 10 | Describe what outcomes were used as the measure(s) of benefit in the evaluation and their relevance for the type of analysis performed. | page 4, line 148 to 170 |
| Measurements of effectiveness | 11a | *Single study-based estimates:* Describe fully the design features of the single effectiveness study and why the single study was a sufficient source of clinical effectiveness data. | page 4, line 148 to 170 |
|  | 11b | *Synthesis-based estimates*: Describe fully the methods used for identification of included studies and synthesis of clinical effectiveness data. | not applicable |
| Measurement and  valuation of preference  based outcomes | 12 | If applicable, describe the population and methods used to elicit preferences for outcomes. | not applicable |
| Estimating resources and  costs | 13a | *Single study-based economic evaluation:* Describe approaches used to estimate resource use associated with the alternative interventions. Describe primary or secondary research methods for valuing each resource item in terms of its unit cost. Describe any adjustments made to approximate to opportunity costs. | page 4, line 171 to 186 |
|  | 13b | *Model-based economic evaluation:* Describe approaches and data sources used to estimate resource use associated with model health states. Describe primary or secondary research methods for valuing each resource item in terms of its unit cost. Describe any adjustments made to approximate to opportunity costs. | not applicable |
| Currency, price date, and  conversion | 14 | Report the dates of the estimated resource quantities and unit costs. Describe methods for adjusting estimated unit costs to the year of reported costs if necessary. Describe methods for converting costs into a common currency base and the exchange rate. | page 4, line 173  page 4, line 180 to 186 |
| Choice of model | 15 | Describe and give reasons for the specific type of decision-analytical model used. Providing a figure to show model structure is strongly recommended. | not applicable |
| Assumptions | 16 | Describe all structural or other assumptions underpinning the decision-analytical model. | page 5, line 187 to 189  page 4, line 153 to 154  page 4, line 158 to 159 |
| Analytical methods | 17 | Describe all analytical methods supporting the evaluation. This could include methods for dealing with skewed, missing, or censored data; extrapolation methods; methods for pooling data; approaches to  validate or make adjustments (such as half cycle  corrections) to a model; and methods for handling population heterogeneity and uncertainty. | page 5-6, line 190-270 |
| **Results** |  |  |  |
| Study parameters | 18 | Report the values, ranges, references, and, if used, probability distributions for all parameters. Report reasons or sources for distributions used to represent uncertainty where appropriate. Providing a table to show the input values is strongly recommended. | not applicable |
| Incremental costs and outcomes | 19 | For each intervention, report mean values for the main categories of estimated costs and outcomes of interest, as well as mean differences between the comparator groups. If applicable, report incremental cost-effectiveness ratios. | page 9, line 348 to 350 |
| Characterising uncertainty | 20a | *Single study-based economic evaluation:* Describe the effects of sampling uncertainty for the estimated incremental cost and incremental effectiveness parameters, together with the impact of methodological assumptions (such as discount rate, study perspective). | page 13, line 405 to 410 |
|  | 20b | *Model-based economic evaluation:* Describe the effects on the results of uncertainty for all input parameters, and uncertainty related to the structure of the model and assumptions. | not applicable |
| Characterising heterogeneity | 21 | If applicable, report differences in costs, outcomes, or cost-effectiveness that can be explained by variations between subgroups of patients with different baseline characteristics or other observed variability in effects that are not reducible by more information. | page 13, line 411 to 423 |
| **Discussion** |  |  |  |
| Study findings, limitations,  generalisability, and current knowledge | 22 | Summarise key study findings and describe how they support the conclusions reached. Discuss limitations and the generalisability of the findings and how the findings fit with current knowledge. | page 13-16, line 424 to 592 |
| **Other** |  |  |  |
| Source of funding | 23 | Describe how the study was funded and the role of the funder in the identification, design, conduct, and reporting of the analysis. Describe other nonmonetary sources of support. | not applicable |
| Conflicts of interest | 24 | Describe any potential for conflict of interest of study contributors in accordance with journal policy. In the absence of a journal policy, we recommend authors comply with International Committee of Medical Journal Editors recommendations. | not applicable |

For consistency, the CHEERS statement checklist format is based on the format of the CONSORT statement checklist.
