# Supplementary material for: Bayesian Regression Model for a Cost-Utility and Cost-Effectiveness Analysis Comparing Punch Grafting Versus Usual Care for the Treatment of Chronic Wounds
Source: Int J Environ Res Public Health. 2020 May 28;17(11):3823. doi: 10.3390/ijerph17113823 (PMC7313055; doi:10.3390/ijerph17113823)
Supplement: Supplementary file 1 [file ijerph-17-03823-s001.zip › Supplementary Table S2. Sensitivity analysis Cost utility.docx]

**Supplementary Table S2.** Sensitivity analysis of CUA-Model: Statistical summary of costs and utility (100,000 simulations MCMC).

|  | **PUNCH** | | **NoPUNCH** | | **Incremental difference** | |
| --- | --- | --- | --- | --- | --- | --- |
|  | **Mean (SD)** | **95% CI** | **Mean (SD)** | **95% CI** | **Mean (SD)** | **95% CI** |
| **Costs -10%** | | | | | | |
| **Costs (€)** | 967 (101) | (816; 1144) | 1563 (188) | (1283; 1894) | 0.63 (0.10) | **(0.48; 0.80)** |
| **Utility (*QALYs*)** | 0.05 (0.02) | (0.02; 0.08) | 0.04 (0.02) | (0.00; 0.07) | 0.02 (0.03) | (-0.03; 0.06) |
| **Costs +10%** | | | | | | |
| **Costs (€)** | 1194 (126.4) | (1005; 1416) | 1942 (237.3) | (1590; 2359) | 0.62 (0.10) | **(0.48; 0.80)** |
| **Utility (*QALYs*)** | 0.05 (0.02) | (0.02; 0.08) | 0.04 (0.02) | (0.00; 0.07) | 0.02 (0.03) | (-0.03; 0.06) |
| **QALYs -10%** | | | | | | |
| **Costs (€)** | 1074 (112.20) | (907; 1271) | 1737 (209.4) | (1426; 2105) | 0.63 (0.10) | **(0.48; 0.80)** |
| **Utility (*QALYs*)** | 0.05 (0.02) | (0.02; 0.08) | 0.03 (0,02) | (-0.00; 0.07) | 0.01 (0.03) | (-0.03; 0.06) |
| **QALYs +10%** | | | | | | |
| **Costs (€)** | 1074 (112.2) | (907; 1271) | 1737 (209.4) | (1426; 2105) | 0.63 (0.10) | **(0.48; 0.80)** |
| **Utility (*QALYs*)** | 0.06 (0.02) | (0.03; 0.09) | 0.04 (0.02) | (0.00; 0.08) | 0.02 (0.03) | (-0.03; 0.07) |
| **QALYs calculated as a difference between utilities** | | | | | | |
| **Costs (€)** | 1074 (112.2) | (906.9; 1271) | 1737 (209.4) | (1426; 2105) | 0.63 (0.10) | **(0.48; 0.80)** |
| **Utility (*QALYs*)** | 0.31 (0.04) | (0.24; 0.37) | 0.23 (0.05) | (0.15; 0.30) | 0.08 (0.06) | (-0.02; 0.18) |
| **Leg ulcers (PUNCH: n=36; NoPUNCH: n=39)** | | | | | | |
| **Costs (€)** | 1203 (139.3) | (997.7; 1450) | 1851 (222.6) | (1522; 2248) | 0.66 (0.10) | **(0.50; 0.84)** |
| **Utility (*QALYs*)** | 0.05 (0.02) | (0.01; 0.09) | 0.03 (0.02) | (-0.00; 0.07) | 0.02 (0.03) | (-0.04; 0.07) |
| **Wound size (26 pairs of patients matched according to their wound size)** | | | | | | |
| **Costs (€)** | 1108 (179.3) | (854.3; 1431) | 1706 (274.6) | (1316; 2201) | 0.66 (0.14) | **(0.46; 0.92)** |
| **Utility (*QALYs*)** | 0.05 (0.03) | (-0.00; 0.10) | 0.04 (0.03) | (-0.01; 0.09) | 0.01 (0.05) | (-0.06; 0.09) |
| **Extreme scenario analysis (worst scenario for PUNCH)** | | | | | | |
| **Costs (€)** | 1181 (123.4) | (998; 1398) | 1563 (188.5) | (1283; 1895) | 0.77 (0.12) | **(0.59; 0.98)** |
| **Utility (*QALYs*)** | 0.05 (0.02) | (0.02; 0.08) | 0.04 (0.02) | (0.00; 0.08) | 0.01 (0.03) | (-0.04; 0.06) |

MCMC: Markov Chain Monte Carlo. QALYs: quality-adjusted life years. CI: Credible Interval. Highlighted: intervals not including the zero value.
