# Supplementary material for: Bayesian Regression Model for a Cost-Utility and Cost-Effectiveness Analysis Comparing Punch Grafting Versus Usual Care for the Treatment of Chronic Wounds
Source: Int J Environ Res Public Health. 2020 May 28;17(11):3823. doi: 10.3390/ijerph17113823 (PMC7313055; doi:10.3390/ijerph17113823)
Supplement: Supplementary file 1 [file ijerph-17-03823-s001.zip › Supplementary Table S3. Sensitivity analysis Cost utility Coefficients.docx]

**Supplementary Table S3.** Sensitivity analysis of CUA-Model: Estimations of the posterior distribution of the β-coefficients and of the probabilities related to the cost-utility analysis (100,000 simulations MCMC).

| ***Costs* -10%** | | **Mean (SD)** | **95% CI** |
| --- | --- | --- | --- |
| **Costs** | β_11_ *intercept* | 6,40 (0.35) | (5.81; 6.97) |
|  | β_12_ *WoundDuration* | 0.01 (0.01) | (-0.01; 0.02) |
|  | β_13_ *WoundLeg* | 0.56 (0.23) | **(0.19; 0.92)** |
|  | β_14_ *WoundSize* | 0.01 (0.00) | (-0.00; 0.01) |
|  | β_15_ *EQ-5D* | 0.12 (0.31) | (-0.40; 0.64) |
|  | β_16_ *Wound-QoL* | 0.09 (0.07) | (-0.03; 0.21) |
|  | β_17_ *Treatment* | -0.48 (0.15) | **(-0.73; -0.22)** |
|  | Costs Ratio (exp β_17_) | 0.63 (0.10) | **(0.48; 0.80)** |
| **Utility** | β_21_ *intercept* | 0.08 (0.07) | (-0.03; 0.19) |
|  | β_22_ *WoundDuration* | -0.00 (0.00) | (-0.00; 0.00) |
|  | β_23_ *WoundLeg* | -0.03 (0.04) | (-0.10; 0.05) |
|  | β_24_ *WoundSize* | -0.00 (0.00) | (-0.00; 0.00) |
|  | β_25_ *EQ-5D* | -0.07 (0.06) | (-0.17; 0.02) |
|  | β_26_ *Wound-QoL* | 0.01 (0.01) | (-0.02; 0.03) |
|  | β_27_ *Treatment* | 0.01 (0.03) | (-0.03; 0.06) |
| **Estimated probability for PUNCH being cheaper** | | 0.9989 (0.0339) | (1.0; 1.0) |
| **Estimated probability for PUNCH being more effective** | | 0.7070 (0.4552) | (0.0; 1.0) |
| **Estimated probability for PUNCH being dominant** | | 0.7063 (0.4555) | (0.0; 1.0) |
| ***Costs* +10%** | | **Mean (SD)** | **95% CI** |
| **Costs** | β_11_ *intercept* | 6.75 (0.36) | (6.16; 7.33) |
|  | β_12_ *WoundDuration* | 0.00 (0.01) | (-0.01; 0.02) |
|  | β_13_ *WoundLeg* | 0.65 (0.23) | **(0.28; 1.03)** |
|  | β_14_ *WoundSize* | 0.00 (0.00) | (-0.00; 0.01) |
|  | β_15_ *EQ-5D* | -0.12 (0.32) | (-0.64; 0.41) |
|  | β_16_ *Wound-QoL* | 0.02 (0.08) | (-0.11; 0.14) |
|  | β_17_ *Treatment* | -0.49 (0.16) | **(-0.74; -0.23)** |
|  | Costs Ratio (exp β_17_) | 0.62 (0.10) | **(0.48; 0.80)** |
| **Utility** | β_21_ *intercept* | 0.09 (0.07) | (-0.02; 0.20) |
|  | β_22_ *WoundDuration* | 0.00 (0.00) | (-0.00; 0.00) |
|  | β_23_ *WoundLeg* | -0.03 (0.04) | (-0.10; 0.04) |
|  | β_24_ *WoundSize* | 0.00 (0.00) | (-0.00; 0.00) |
|  | β_25_ *EQ-5D* | -0.08 (0.06) | (-0.18; 0.02) |
|  | β_26_ *Wound-QoL* | 0.01 (0.01) | (-0.02; 0.03) |
|  | β_27_ *Treatment* | 0.02 (0.03) | (-0.03; 0.06) |
| **Estimated probability for PUNCH being cheaper** | | 0.9989 (0.0334) | (1.0; 1.0) |
| **Estimated probability for PUNCH being more effective** | | 0.7067 (0.4553) | (0.0; 1.0) |
| **Estimated probability for PUNCH being dominant** | | 0.7061 (0.4555) | (0.0; 1.0) |
| ***QALYs* -10%** | | **Mean (SD)** | **95% CI** |
| **Costs** | β_11_ *intercept* | 6,40 (0.35) | (5.81; 6.97) |
|  | β_12_ *WoundDuration* | 0.01 (0.01) | (-0.01; 0.02) |
|  | β_13_ *WoundLeg* | 0.56 (0.23) | **(0.19; 0.92)** |
|  | β_14_ *WoundSize* | 0.01 (0.00) | (-0.00; 0.01) |
|  | β_15_ *EQ-5D* | 0.12 (0.31) | (-0.40; 0.64) |
|  | β_16_ *Wound-QoL* | 0.09 (0.07) | (-0.03; 0.21) |
|  | β_17_ *Treatment* | -0.48 (0.15) | **(-0.73; -0.22)** |
|  | Costs Ratio (exp β_17_) | 0.63 (0.10) | **(0.48; 0.80)** |
| **Utility** | β_21_ *intercept* | 0.08 (0.07) | (-0.03; 0.19) |
|  | β_22_ *WoundDuration* | -0.00 (0.00) | (-0.00; 0.00) |
|  | β_23_ *WoundLeg* | -0.03 (0.04) | (-0.10; 0.05) |
|  | β_24_ *WoundSize* | -0.00 (0.00) | (-0.00; 0.00) |
|  | β_25_ *EQ-5D* | -0.07 (0.06) | (-0.17; 0.02) |
|  | β_26_ *Wound-QoL* | 0.01 (0.01) | (-0.02; 0.03) |
|  | β_27_ *Treatment* | 0.01 (0.03) | (-0.03; 0.06) |
| **Estimated probability for PUNCH being cheaper** | | 0.9989 (0.0336) | (1.0; 1.0) |
| **Estimated probability for PUNCH being more effective** | | 0.6893 (0.4628) | (0.0; 1.0) |
| **Estimated probability for PUNCH being dominant** | | 0.6886 (0.4630) | (0.0; 1.0) |
| ***QALYs* +10%** | | **Mean (SD)** | **95% CI** |
| **Costs** | β_11_ *intercept* | 6.40 (0.35) | (5.82; 6.97) |
|  | β_12_ *WoundDuration* | 0.01 (0.01) | (-0.01; 0.02) |
|  | β_13_ *WoundLeg* | 0.56 (0.23) | **(0.19; 0.92)** |
|  | β_14_ *WoundSize* | 0.01 (0.00) | **(0.00; 0.01)** |
|  | β_15_ *EQ-5D* | 0.12 (0.31) | (-0.39; 0.64) |
|  | β_16_ *Wound-QoL* | 0.09 (0.07) | (-0.03; 0.21) |
|  | β_17_ *Treatment* | -0.48 (0.15) | **(-0.73; -0.22)** |
|  | Costs Ratio (exp β_17_) | 0.63 (0.10) | **(0.48; 0.80)** |
| **Utility** | β_21_ *intercept* | 0.10 (0.07) | (-0.01; 0.21) |
|  | β_22_ *WoundDuration* | 0.00 (0.00) | (-0.00; 0.00) |
|  | β_23_ *WoundLeg* | -0.03 (0.04) | (-0.10; 0.04) |
|  | β_24_ *WoundSize* | 0.00 (0.00) | (-0.00; 0.00) |
|  | β_25_ *EQ-5D* | -0.09 (0.06) | (-0.19; 0.01) |
|  | β_26_ *Wound-QoL* | 0.01 (0.01) | (-0.02; 0.03) |
|  | β_27_ *Treatment* | 0.02 (0.03) | (-0.03; 0.07) |
| **Estimated probability for PUNCH being cheaper** | | 0.9988 (0.0340) | (1.0; 1.0) |
| **Estimated probability for PUNCH being more effective** | | 0.7228 (0.4476) | (0.0; 1.0) |
| **Estimated probability for PUNCH being dominant** | | 0.7221 (0.4480) | (0.0; 1.0) |
| ***QALYs* calculated as a difference between utilities** | | **Mean (SD)** | **95% CI** |
| **Costs** | β_11_ *intercept* | 6.40 (0.35) | (5.82; 6.97) |
|  | β_12_ *WoundDuration* | 0.01 (0.01) | (-0.01, 0.02) |
|  | β_13_ *WoundLeg* | 0.56 (0.23) | **(0.19; 0.92)** |
|  | β_14_ *WoundSize* | 0.01 (0.00) | **(0.00; 0.01)** |
|  | β_15_ *EQ-5D* | 0.12 (0.31) | (-0.39; 0.64) |
|  | β_16_ *Wound-QoL* | 0.09 (0.07) | (-0.03; 0.21) |
|  | β_17_ *Treatment* | -0.48 (0.15) | **(-0.73; -0.22)** |
|  | Costs Ratio (exp β_17_) | 0.63 (0.10) | **(0.48; 0.80)** |
| **Utility** | β_21_ *intercept* | 0.47 (0.14) | (0.24; 0.70) |
|  | β_22_ *WoundDuration* | -0.00 (0.00) | (-0.01; 0.00) |
|  | β_23_ *WoundLeg* | -0.12 (0.09) | (-0.27; 0.03) |
|  | β_24_ *WoundSize* | 0.00 (0.00) | (-0.00; 0.00) |
|  | β_25_ *EQ-5D* | -0.51 (0.13) | **(-0.72; -0.30)** |
|  | β_26_ *Wound-QoL* | 0.06 (0.03) | **(0.01; 0.11)** |
|  | β_27_ *Treatment* | 0.08 (0.06) | (-0.02; 0.18) |
| **Estimated probability for PUNCH being cheaper** | | 0.9988 (0.0343) | (1.0; 1.0) |
| **Estimated probability for PUNCH being more effective** | | 0.9030 (0.2959) | (0.0; 1.0) |
| **Estimated probability for PUNCH being dominant** | | 0.9021 (0.2972) | (0.0; 1.0) |
| **Leg ulcers (PUNCH: n=36; NoPUNCH n=39)** | | **Mean (SD)** | **95% CI** |
| **Costs** | β_11_ *intercept* | 6.94 (0.31) | (6.43; 7.45) |
|  | β_12_ *WoundDuration* | 0.00 (0.01) | (-0.01; 0.02) |
|  | β_14_ *WoundSize* | 0.01 (0.00) | **(0.00; 0.01)** |
|  | β_15_ *EQ-5D* | 0.13 (0.34) | (-0.42; 0.68) |
|  | β_16_ *Wound-QoL* | 0.09 (0.08) | (-0.04, 0.21) |
|  | β_17_ *Treatment* | -0.43 (0.16) | **(-0.68; -0.17)** |
|  | Costs Ratio (exp β_17_) | 0.66 (0.10) | **(0.50; 0.84)** |
| **Utility** | β_21_ *intercept* | 0.05 (0.06) | (-0.06; 0.16) |
|  | β_22_ *WoundDuration* | 0.00 (0.00) | (-0.00; 0.00) |
|  | β_24_ *WoundSize* | -0.00 (0.00) | (-0.00; 0.00) |
|  | β_25_ *EQ-5D* | -0.07 (0.07) | (-0.18; 0.05) |
|  | β_26_ *Wound-QoL* | 0.01 (0.02) | (-0.02; 0.04) |
|  | β_27_ *Treatment* | 0.02 (0.03) | (-0.04; 0.07) |
| **Estimated probability for PUNCH being cheaper** | | 0.9969 (0.0557) | (1.0; 1.0) |
| **Estimated probability for PUNCH being more effective** | | 0.6851 (0.4645) | ((0.0; 1.0) |
| **Estimated probability for PUNCH being dominant** | | 0.6851 (0.4645) | (0.0; 1.0) |
| **Wound size (26 pairs of patients matched according to their wound size)** | | **Mean (SD)** | **95% CI** |
| **Costs** | β_11_ *intercept* | 6.14 (0.53) | (5.27; 7.02) |
|  | β_12_ *WoundDuration* | 0.00 (0.01) | (-0.02; 0.02) |
|  | β_13_ *WoundLeg* | 0.85 (0.36) | **(0.25; 1.44)** |
|  | β_15_ *EQ-5D* | 0.21 (0.44) | (-0.51; 0.93) |
|  | β_16_ *Wound-QoL* | 0.07 (0.10) | (-0.10: 0.24) |
|  | β_17_ *Treatment* | -0.43 (0.21) | **(-0.78; -0.08)** |
|  | Costs Ratio (exp β_17_) | 0.66 (0.14) | **(0.46; 0.92)** |
| **Utility** | β_21_ *intercept* | 0.09 (0.12) | (-0.10; 0.28) |
|  | β_22_ *WoundDuration* | 0.00 (0.00) | (-0.00; 0.00) |
|  | β_23_ *WoundLeg* | -0.05 (0.08) | (-0.17; 0.08) |
|  | β_25_ *EQ-5D* | -0.06 (0.09) | (-0.22; 0.09) |
|  | β_26_ *Wound-QoL* | 0.01 (0.02) | (-0.03; 0.05) |
|  | β_27_ *Treatment* | 0.01 (0.05) | (-0.06; 0.09) |
| **Estimated probability for PUNCH being cheaper** | | 0.9779 (0.1469) | (1.0; 1.0) |
| **Estimated probability for PUNCH being more effective** | | 0.6041 (0.4890) | (0.0; 1.0) |
| **Estimated probability for PUNCH being dominant** | | 0.5917 (0.4915) | (0.0; 1.0) |
| **Extreme scenario analysis (worst scenario for PUNCH)** | | **Mean (SD)** | **95% CI** |
| **Costs** | β_11_ *intercept* | 6.23 (0.35) | (5.71; 6.87) |
|  | β_12_ *WoundDuration* | 0.01 (0.01) | (-0.01; 0.02) |
|  | β_13_ *WoundLeg* | 0.56 (0.23) | **(0.19; 0.92)** |
|  | β_14_ *WoundSize* | 0.01 (0.00) | (-0.00; 0.02) |
|  | β_15_ *EQ-5D* | 0.12 (0.31) | (-0.40; 0.64) |
|  | β_16_ *Wound-QoL* | 0.09 (0.07) | (-0.03; 0.21) |
|  | β_17_ *Treatment* | -0.28 (0.15) | **(-0.53; -0.02)** |
|  | Costs Ratio (exp β_17_) | 0.77 (0.12) | **(0.59; 0.98)** |
| **Utility** | β_21_ *intercept* | 0.09 (0.07) | (-0.02; 0.20) |
|  | β_22_ *WoundDuration* | -0.00 (0.00) | (-0.00; 0.00) |
|  | β_23_ *WoundLeg* | -0.02 (0.04) | (-0.10; 0.04) |
|  | β_24_ *WoundSize* | 0.00 (0.00) | (-0.00; 0.00) |
|  | β_25_ *EQ-5D* | -0.08 (0.06) | (-0.18; 0.02) |
|  | β_26_ *Wound-QoL* | 0.01 (0.01) | (-0.02; 0.03) |
|  | β_27_ *Treatment* | 0.01 (0.03) | (-0.04; 0.06) |
| **Estimated probability for PUNCH being cheaper** | | 0.9639 (0.1866) | (1.0; 1.0) |
| **Estimated probability for PUNCH being more effective** | | 0.5916 (0.4915) | (0.0; 1.0) |
| **Estimated probability for PUNCH being dominant** | | 0.5726 (0.4947) | (0.0; 1.0) |

EQ-5D: generic questionnaire used to assess HRQoL. Wound-QoL: specific questionnaire measuring HRQoL in patients suffering from chronic wounds. CI: Credible Interval. Highlighted: intervals not including the zero value.
