# Supplementary material for: Bayesian Regression Model for a Cost-Utility and Cost-Effectiveness Analysis Comparing Punch Grafting Versus Usual Care for the Treatment of Chronic Wounds
Source: Int J Environ Res Public Health. 2020 May 28;17(11):3823. doi: 10.3390/ijerph17113823 (PMC7313055; doi:10.3390/ijerph17113823)
Supplement: Supplementary file 1 [file ijerph-17-03823-s001.zip › Supplementary Table S4. Sensitivity analysis Cost effectiveness.docx]

**Supplementary Table S4.** Sensitivity analysis of CEA-Model: Statistical summary of costs and effectiveness (100,000 simulations MCMC).

|  | **PUNCH** | | **NoPUNCH** | | **Incremental difference** | |
| --- | --- | --- | --- | --- | --- | --- |
|  | **Mean (SD)** | **95% CI** | **Mean (SD)** | **95% CI** | **Mean (SD)** | **95% CI** |
| **Costs -10%** | | | | | | |
| **Costs (€)** | 968 (100.6) | (817.3; 1144) | 1563 (186.9) | (1285; 1892) | 0.63 (0.10) | (0.48; 0.80) |
| **Effectiveness (*wound-free period*)** | 34.16 (3.35) | (28.64; 39.63) | 26.98 (3.93) | (20.53; 22.42) | 7.18 (5.30) | (-1.57; 15.89) |
| **Costs +10%** | | | | | | |
| **Costs (€)** | 1182 (122.9) | (998.8; 1398) | 1910 (228.5) | (1570; 2313) | 0.63 (0.10) | **(0.48; 0.80)** |
| **Effectiveness (*wound-free period*)** | 34.16 (3.35) | (26.85; 39.63) | 26.98 (3.93) | (20.53; 22.42) | 7.18 (5.30) | (-1.57; 15.89) |
| **Wound-free period -10%** | | | | | | |
| **Costs (€)** | 1075 (111.7) | (908.1; 1271) | 1737 (207.7) | (1428; 2102) | 0.63 (0.10) | **(0.48; 0.80)** |
| **Effectiveness (*wound-free period*)** | 30.77 (3.02) | (25.80; 35.69) | 24.28 (3.54) | (18.48; 30.08) | 6.49 (4.78) | (-1.39; 14.32) |
| **Wound-free period +10%** | | | | | | |
| **Costs (€)** | 1075 (111.7) | (908.1; 1271) | 1737 (207.7) | (1428; 2102) | 0.63 (0.10) | **(0.48; 0.80)** |
| **Effectiveness (*wound-free period*)** | 37.66 (3.69) | (31.59; 43.68) | 29.68 (4.33) | (22.58; 36.77) | 7.98 (5.84) | (-1.66; 17.56) |
| **Leg ulcers (PUNCH: n = 36; NoPUNCH: n = 39)** | | | | | | |
| **Costs (€)** | 1204 (140.1) | (997.9; 1452) | 1852 (223.1) | (1523; 2248) | 0.66 (0.10) | **(0.50; 0.84)** |
| **Effectiveness (*wound-free period*)** | 30.32 (3.82) | (24.04; 36.57) | 23.87 (3.98) | (17.33; 30.4) | 6.44 (5.54) | (-2.64; 15.57) |
| **Wound size (26 pairs of patients matched according to their wound size)** | | | | | | |
| **Costs (€)** | 1108 (179.6) | (855; 1432) | 1707 (276.3) | (1318; 2205) | 0.66 (0.14) | **(0.46; 0.92)** |
| **Effectiveness (*wound-free period*)** | 35.53 (5.27) | (26.84; 44.12) | 30.01 (5.27) | (21.34; 38.67) | 5.52 (7.70) | (-7.15; 18.1) |
| **Extreme scenario analysis (worst scenario for PUNCH)** | | | | | | |
| **Costs (€)** | 1182 (122.9) | (999; 1398) | 1563 (187) | (1285; 1892) | 0.77 (0.12) | **(0.59; 0.97)** |
| **Effectiveness (*wound-free period*)** | 30.64 (3.28) | (25.24; 35.99) | 29.16 (3,84) | (22.85; 35.48) | 1.47 (5.19) | (-7.08; 9.98) |

MCMC: Markov Chain Monte Carlo. CI: Credible Interval. Highlighted: intervals not including the zero value.
