# Supplementary material for: Bayesian Regression Model for a Cost-Utility and Cost-Effectiveness Analysis Comparing Punch Grafting Versus Usual Care for the Treatment of Chronic Wounds
Source: Int J Environ Res Public Health. 2020 May 28;17(11):3823. doi: 10.3390/ijerph17113823 (PMC7313055; doi:10.3390/ijerph17113823)
Supplement: Supplementary file 1 [file ijerph-17-03823-s001.zip › Supplementary Table S5. Sensitivity analysis Cost effectiveness Coefficients.docx]

**Supplementary Table S5.** Sensitivity analysis of CEA-Model: Estimations of the posterior distribution of the β-coefficients and of the probabilities related to the cost-effectiveness analysis (100,000 simulations MCMC).

| ***Costs* -10%** | | **Mean (SD)** | **95% CI** |
| --- | --- | --- | --- |
| **Costs** | β_11_ *intercept* | 6.29 (0.35) | (5.71; 6.87) |
|  | β_12_ *WoundDuration* | 0.01 (0.01) | (-0.01; 0.02) |
|  | β_13_ *WoundLeg* | 0.55 (0.23) | **(0.18; 0.92)** |
|  | β_14_ *WoundSize* | 0.01 (0.00) | **(0.00; 0.01)** |
|  | β_15_ *EQ-5D* | 0.12 (0.31) | (-0.39; 0.64) |
|  | β_16_ *Wound-QoL* | 0.09 (0.07) | (-0.03; 0.21) |
|  | β_17_ *Treatment* | -0.48 (0.15) | **(-0.73; -0.23)** |
|  | Costs Ratio (exp β_17_) | 0.63 (0.10) | **(0.48; 0.80)** |
| **Effectiveness** | β_31_ *intercept* | 57.54 (12.25) | (37.41; 77.62) |
|  | β_32_ *WoundDuration* | -0.39 (0.25) | (-0.80; 0.02) |
|  | Β_33_ *WoundLeg* | -18.66 (7.86) | **(-31.56; -5.76)** |
|  | β_34_ *WoundSize* | -0.35 (0.14) | **(-0.57; -0.12)** |
|  | β_35_ *EQ-5D* | 3.44 (10.91) | (-14.49; 21.33) |
|  | β_36_ *Wound-QoL* | -4.06 (2.60) | (-8.35; 0.22) |
|  | β_37_ *Treatment* | 7.18 (5.30) | (-1.57; 15.89) |
| **Estimated probability for PUNCH being cheaper** | | 0.9988 (0.035) | (1.0; 1.0) |
| **Estimated probability for PUNCH being more effective** | | 0.9129 (0.2820) | (0.0; 1.0) |
| **Estimated probability for PUNCH being dominant** | | 0.9128 (0.2821) | (0.0; 1.0) |
| ***Costs* +10%** | | **Mean (SD)** | **95% CI** |
| **Costs** | β_11_ *intercept* | 6.49 (0.35) | (5.91; 7.07) |
|  | β_12_ *WoundDuration* | 0.01 (0.01) | (-0.01; 0.02) |
|  | β_13_ *WoundLeg* | 0.56 (0.23) | **(0.18; 0.92)** |
|  | β_14_ *WoundSize* | 0.01 (0.00) | **(0.00; 0.01)** |
|  | β_15_ *EQ-5D* | 0.12 (0.31) | (-0.39; 0.64) |
|  | β_16_ *Wound-QoL* | 0.09 (0.07) | (-0.03; 0.21) |
|  | β_17_ *Treatment* | -0.48 (0.15) | **(-0.73; -0.23)** |
|  | Costs Ratio (exp β_17_) | 0.63 (0.10) | **(0.48; 0.80)** |
| **Effectiveness** | β_31_ *intercept* | 57.54 (12.25) | (37.41; 77.62) |
|  | β_32_ *WoundDuration* | -0.39 (0.25) | (-0.80; 0.02) |
|  | Β_33_ *WoundLeg* | -18.66 (7.86) | **(-31.56; -5.76)** |
|  | β_34_ *WoundSize* | -0.35 (0.14) | **(-0.57; -0.12)** |
|  | β_35_ *EQ-5D* | 3.44 (10.91) | (-14.49; 21.33) |
|  | β_36_ *Wound-QoL* | -4.06 (2.60) | (-8.35; 0.22) |
|  | β_37_ *Treatment* | 7.18 (5.30) | (-1.57; 15.89) |
| **Estimated probability for PUNCH being cheaper** | | 0.9988 (0.0346) | (1.0; 1.0) |
| **Estimated probability for PUNCH being more effective** | | 0.9129 (0.2821) | (0.0; 1.0) |
| **Estimated probability for PUNCH being dominant** | | 0.9128 (0.2821) | (0.0; 1.0) |
| ***Wound-free period* -10%** | | **Mean (SD)** | **95% CI** |
| **Costs** | β_11_ *intercept* | 6.39 (0.35) | (5.82; 6.97) |
|  | β_12_ *WoundDuration* | 0.01 (0.01) | (-0.01; 0.02) |
|  | β_13_ *WoundLeg* | 0.55 (0.23) | **(0.18; 0.92)** |
|  | β_14_ *WoundSize* | 0.01 (0.00) | (-0.00; 0.01) |
|  | β_15_ *EQ-5D* | 0.12 (0.31) | (-0.39; 0.64) |
|  | β_16_ *Wound-QoL* | 0.09 (0.07) | (-0.03; 0.21) |
|  | β_17_ *Treatment* | -0.48 (0.15) | **(-0.73; -0.23)** |
|  | Costs Ratio (exp β_17_) | 0.63 (0.10) | **(0.48; 0.80)** |
| **Effectiveness** | β_31_ *intercept* | 51.66 (11.03) | (33.54; 69.74) |
|  | β_32_ *WoundDuration* | -0.36 (0.22) | (-0.72; 0.01) |
|  | Β_33_ *WoundLeg* | -16.75 (7.07) | **(-28.37; -5.14)** |
|  | β_34_ *WoundSize* | -0.31 (0.12) | **(-0.51; -0.11)** |
|  | β_35_ *EQ-5D* | 3.26 (9.82) | (-12.89; 19.36) |
|  | β_36_ *Wound-QoL* | -3.64 (2.34) | (-7.49; 0.22) |
|  | β_37_ *Treatment* | 6.49 (4.78) | (-1.39; 14.32) |
| **Estimated probability for PUNCH being cheaper** | | 0.9988 (0.0346) | (1.0; 1.0) |
| **Estimated probability for PUNCH being more effective** | | 0.9136 (0.2809) | (0.0; 1.0) |
| **Estimated probability for PUNCH being dominant** | | 0.9136 (0.2809) | (0.0; 1.0) |
| ***Wound-free period* +10%** | | **Mean (SD)** | **95% CI** |
| **Costs** | β_11_ *intercept* | 6.40 (0.35) | (5.82; 6.97) |
|  | β_12_ *WoundDuration* | 0.01 (0.01) | (-0.01; 0.02) |
|  | β_13_ *WoundLeg* | 0.55 (0.23) | **(0.18; 0.92)** |
|  | β_14_ *WoundSize* | 0.01 (0.00) | **(0.00; 0.01)** |
|  | β_15_ *EQ-5D* | 0.12 (0.31) | (-0.39; 0.64) |
|  | β_16_ *Wound-QoL* | 0.01 (0.07) | (-0.03; 0.21) |
|  | β_17_ *Treatment* | -0.48 (0.15) | **(-0.73; -0.23)** |
|  | Costs Ratio (exp β_17_) | 0.63 (0.10) | **(0.48; 0.80)** |
| **Effectiveness** | β_31_ *intercept* | 63.28 (13.49) | (42.12; 85.39) |
|  | β_32_ *WoundDuration* | -0.43 (0.27) | (-0.88; 0.02) |
|  | Β_33_ *WoundLeg* | -20.51 (8.65) | **(-34.71; -6.31)** |
|  | β_34_ *WoundSize* | -0.38 (0.15) | **(-0.63; -0.13)** |
|  | β_35_ *EQ-5D* | 3.86 (12.01) | (-15.89; 23.55) |
|  | β_36_ *Wound-QoL* | -4.49 (2.86) | (-9.20; 0.23) |
|  | β_37_ *Treatment* | 7.98 (5.84) | (-1.66; 17.56) |
| **Estimated probability for PUNCH being cheaper** | | 0.9988 (0.0346) | (1.0; 1.0) |
| **Estimated probability for PUNCH being more effective** | | 0.9148 (0.2791) | (0.0; 1.0) |
| **Estimated probability for PUNCH being dominant** | | 0.9148 (0.2791) | (0.0; 1.0) |
| **Leg ulcers (PUNCH: n=36; NoPUNCH n=39)** | | **Mean (SD)** | **95% CI** |
| **Costs** | β_11_ *intercept* | 6.94 (0.31) | (6.43; 7.45) |
|  | β_12_ *WoundDuration* | 0.00 (0.01) | (-0.01; 0.01) |
|  | β_14_ *WoundSize* | 0.01 (0.00) | (-0.00; 0.01) |
|  | β_15_ *EQ-5D* | 0.13 (0.34) | (-0.43; 0.68) |
|  | β_16_ *Wound-QoL* | 0.09 (0.08) | (-0.04; 0.21) |
|  | β_17_ *Treatment* | -0.43 (0.16) | **(-0.69; -0.17)** |
|  | Costs Ratio (exp β_17_) | 0.66 (0.10) | **(0.50; 0.84)** |
| **Effectiveness** | β_31_ *intercept* | 38.91 (10.96) | (20.9; 56.86) |
|  | β_32_ *WoundDuration* | -0.36 (0.25) | (-0.77; 0.06) |
|  | β_34_ *WoundSize* | -0.36 (0.14) | **(-0.59; -0.13)** |
|  | β_35_ *EQ-5D* | 2.64 (11.97) | (-17.12; 22.22) |
|  | β_36_ *Wound-QoL* | -3.79 (2.72) | (-8.27; 0.67) |
|  | β_37_ *Treatment* | 6.44 (5.54) | (-2.64; 15.57) |
| **Estimated probability for PUNCH being cheaper** | | 0.9965 (0.0591) | (1.0; 1.0) |
| **Estimated probability for PUNCH being more effective** | | 0.8794 (0.3256) | (0.0; 1.0) |
| **Estimated probability for PUNCH being dominant** | | 0.8794 (0.3257) | (0.0; 1.0) |
| **Wound size (26 pairs of patients matched according to their wound size)** | | **Mean (SD)** | **95% CI** |
| **Costs** | β_11_ *intercept* | 6.15 (0.54) | (5.27; 7.02) |
|  | β_12_ *WoundDuration* | -0.00 (0.01) | (-0.02; 0.02) |
|  | β_13_ *WoundLeg* | 0.84 (0.36) | **(0.24; 1.44)** |
|  | β_15_ *EQ-5D* | 0.21 (0.44) | (-0.51; 0.93) |
|  | β_16_ *Wound-QoL* | 0.07 (0.10) | (-0.10; 0.24) |
|  | β_17_ *Treatment* | -0.43 (0.21) | **(-0.78; -0.08)** |
|  | Costs Ratio (exp β_17_) | 0.66 (0.14) | **(0.46; 0.92)** |
| **Effectiveness** | β_31_ *intercept* | 57.77 (19.26) | (26.24; 89.39) |
|  | β_32_ *WoundDuration* | -0.13 (0.42) | (-0.82; 0.57) |
|  | Β_33_ *WoundLeg* | -19.3 (13.03) | (-40.62; 2.08) |
|  | β_35_ *EQ-5D* | 1.16 (15.7) | (-24.55; 26.88) |
|  | β_36_ *Wound-QoL* | -4.63 (3.72) | (-10.71; 1.47) |
|  | β_37_ *Treatment* | 5.52 (7.70) | (-7.15; 18.1) |
| **Estimated probability for PUNCH being cheaper** | | 0.9773 (0.1489) | (1.0; 1.0) |
| **Estimated probability for PUNCH being more effective** | | 0.7652 (0.4239) | (0.0; 1.0) |
| **Estimated probability for PUNCH being dominant** | | 0.7647 (0.4242) | (0.0; 1.0) |
| **Extreme scenario analysis (worst scenario for PUNCH)** | | **Mean (SD)** | **95% CI** |
| **Costs** | β_11_ *intercept* | 6.29 (0.35) | (5.71; 6.87) |
|  | β_12_ *WoundDuration* | 0.01 (0.01) | (-0.01; 0.02) |
|  | β_13_ *WoundLeg* | 0.56 (0.23) | **(0.18; 0.92)** |
|  | β_14_ *WoundSize* | 0.01 (0.00) | (-0.00; 0.01) |
|  | β_15_ *EQ-5D* | 0.12 (0.31) | (-0.39; 0.64) |
|  | β_16_ *Wound-QoL* | 0.09 (0.07) | (-0.03; 0.21) |
|  | β_17_ *Treatment* | -0.28 (0.15) | **(-0.53, -0.03)** |
|  | Costs Ratio (exp β_17_) | 0.77 (0.12) | **(0.59; 0.97)** |
| **Effectiveness** | β_31_ *intercept* | 57.87 (11.99) | (38.2; 77.53) |
|  | β_32_ *WoundDuration* | -0.36 (0.24) | (-0.76; 0.04) |
|  | Β_33_ *WoundLeg* | -16.33 (7.69) | **(-28.95; -3.71)** |
|  | β_34_ *WoundSize* | -0.35 (0.14) | **(-0.57; -0.13)** |
|  | β_35_ *EQ-5D* | 3.93 (10.67) | (-13.61; 21.43) |
|  | β_36_ *Wound-QoL* | -4.35 (2.54) | **(-8.54; -0.16)** |
|  | β_37_ *Treatment* | 1.47 (5.19) | (-7.08; 9.98) |
| **Estimated probability for PUNCH being cheaper** | | 0.9642 (0.1858) | (1.0; 1.0) |
| **Estimated probability for PUNCH being more effective** | | 0.6135 (0.4869) | (0.0; 1.0) |
| **Estimated probability for PUNCH being dominant** | | 0.6130 (0.4871) | (0.0; 1.0) |

EQ-5D: generic questionnaire used to assess HRQoL. Wound-QoL: specific questionnaire measuring HRQoL in patients suffering from chronic wounds. CI: Credible Interval. Highlighted: intervals not including the zero value.
